# The impact of coronary artery bypass grafting added to aortic valve replacement on long-term outcomes in octogenarian patients: a reconstructed time-to-event meta-analysis

**DOI:** 10.1093/icvts/ivac164

**Published:** 2022-06-20

**Authors:** Alan Gallingani, Stefano D’Alessandro, Gurmeet Singh, Daniel Hernandez-Vaquero, Mevlüt Çelik, Evelina Ceccato, Francesco Nicolini, Francesco Formica

**Affiliations:** Cardiac Surgery Unit, Parma University Hospital, Parma, Italy; Cardiac Surgery Unit, San Gerardo Hospital, Monza, Italy; Department of Critical Care Medicine and Division of Cardiac Surgery, Mazankowski, Alberta Heart Institute, University of Alberta, Edmonton, Canada; Cardiac Surgery Department, Hospital Universitario Central de Asturias, Oviedo, Spain; Department of Cardiothoracic Surgery, Erasmus University Medical Center, Rotterdam, Netherlands; Medical Library, University of Parma, Parma, Italy; Medical Library, University of Parma, Parma, Italy; Department of Medicine and Surgery, University of Parma, Parma, Italy; Medical Library, University of Parma, Parma, Italy; Department of Medicine and Surgery, University of Parma, Parma, Italy

**Keywords:** Octogenarians, Surgical aortic valve replacement, Coronary artery bypass grafting, Meta-analisys

## Abstract

**Figure:**
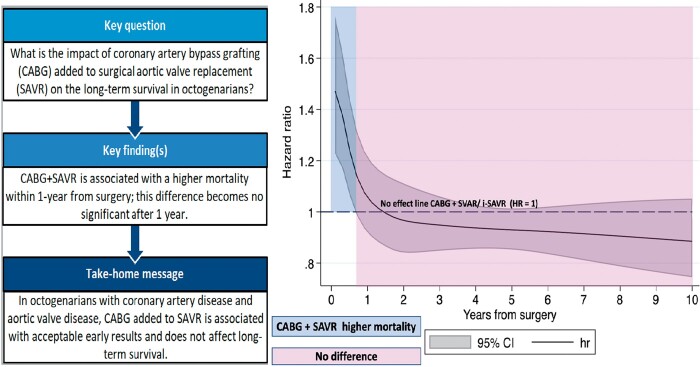

The long-term results in studies comparing octogenarian patients who received either isolated surgical aortic valve replacement (i-SAVR) or coronary artery bypass grafting (CABG) in addition to SAVR are still debated. We performed a reconstructed time-to-event data meta-analysis of studies comparing i-SAVR and CABG+SAVR to evaluate the impact of CABG and to analyse the time-varying effects on long-term outcome. We performed a systematic review of the literature from January 2000 through November 2021, including studies comparing i-SAVR and CABG+SAVR, which reported at least 3-year follow-up and that plotted Kaplan–Meier curves of overall survival. The primary endpoint was overall long-term survival; secondary endpoints were in-hospital/30-day mortality and postoperative outcomes. The pooled hazard ratio (HR) and odds ratio) with 95% confidence interval (CI) were calculated for primary and secondary endpoints, respectively. Random-effect model was used in all analyses. Sixteen retrospective studies were included (5382 patients, i-SAVR = 2568 and CABG+SAVR = 2814). I-SAVR showed a lower incidence of in-hospital mortality compared to CABG+SAVR (odds ratio = 0.73; 95% CI= 0.60–0.89; *P* = 0.002). Landmark analyses showed a significantly higher all-cause mortality within 1 year from surgery in CABG+SAVR (HR = 1.17; 95% CI = 1.01–1.36; *P* = 0.03); after 1 year, no significant difference was observed (HR = 0.95; 95% CI = 0.87–1.04; *P* = 0.35). Landmark analysis was confirmed by time-varying trend of HR. Late survival of octogenarians did not differ significantly between the 2 interventions. Interestingly, CABG added to SAVR was associated with both higher in-hospital and within 1-year mortality after surgery, whereas this difference was statistically non-significant at long-term follow-up.

## INTRODUCTION

Aortic valve stenosis (AS) and coronary artery disease (CAD) are the most common cardiac diseases in the aged population, and as the octogenarian population will rise in the coming decades, there will also be a concomitant increase in patients with AS and CAD. Surgical aortic valve replacement (SAVR) combined with coronary artery bypass grafting (CABG) is affected by a higher procedural risk compared with isolated SAVR (i-SAVR). While isolated AS could be addressed with transcatheter aortic valve implantation (TAVI) even in octogenarians with intermediate or low risk [1], the TAVI in combination with percutaneous coronary intervention (PCI) is not still a fully accepted procedure, especially for those patients with extensive and heavily calcified CAD. While in-hospital mortality is increased by the concomitant CABG surgery, as recognized by EuroSCORE and Society of Thoracic Surgeon score, the impact on long-term outcomes is debated. Some studies have reported acceptable and comparable long-term results in those patients who received either i-SAVR or CABG combined with SAVR [2, 3], whereas other authors have reported conflicting outcomes, with some studies showing better long-term survival in i-SAVR patients [4] while others reporting longer-term benefit in CABG+SAVR patients [5, 6]. To the best of our knowledge, no randomized control trials or meta-analyses related to the impact on long-term of CABG added to SAVR in octogenarians are available. To address this gap of knowledge, we have performed this systematic review and study-level meta-analysis with the best available evidence, to compare long-term survival of i-SAVR with CABG+SAVR in patients older than 80 years of age, by reconstructing the time-to-event data and focusing on the variation of the hazard ratio (HR) over time.

## MATERIALS AND METHODS

### Ethics statement

The local Ethical Committee waived to review the study because no individual patient data were used for the analysis.

### Systematic review of the literature, search strategy and eligibility criteria

We performed a comprehensive review of relevant studies published between 1 January 2000 and 30 November 2021. Three electronic databases, PubMed, Cochrane Central Register of Controlled Trials (CENTRAL) and EMBASE, were queried to search for studies. A medical librarian (E.C.) implemented the queries through the aforementioned electronic databases. Search terms used alone or in combination included ‘elderly patients’, ‘very elderly’, ‘octogenarians’, ‘80 years old’, ‘surgical aortic valve replacement’, ‘coronary artery bypass grafting’, ‘long-term results’, ‘long-term outcomes’ and ‘long-term survival’. Moreover, the references list of the retrieved articles was used to complete the search. The search algorithm is reported in Supplementary Material, Table S1.

PICOS format (Population; Intervention; Comparison; Outcomes; Studies) was considered for literature search and review as follows:

Population: patients with isolated aortic valve disease or associated with CAD; Intervention: i-SAVR; Comparison: CABG combined with SAVR; Outcomes: long-term survival; Studies: randomized control trials, prospective and retrospective observational studies.

Screening and selection of relevant studies were based on the following inclusion criteria: (i) studies that compared i-SAVR or CABG+SVAR; (ii) patients had to be older than 80 years; (iii) long-term follow-up of at least 3 years comparing the 2 interventions; (iv) evidence of figures showing the Kaplan–Meier curves comparing the 2 interventions; (v) outcomes studied included any of the following postoperative complications: early mortality, use of intra-aortic balloon pump (IABP), cerebrovascular events (CVE), new onset of postoperative atrial fibrillation (POAF), acute kidney injury (AKI), re-thoracotomy for bleeding/tamponade and prolonged mechanical ventilation (PMV; >24 h). Studies including other associated cardiac procedures were excluded. Moreover, studies published in languages other than English, commentaries, letters, case reports, systematic reviews and meta-analyses were excluded.

This systematic review and meta-analysis were conducted based on the Preferred Reporting Items for Systematic Reviews and Meta-Analyses (PRISMA) checklist [7] (Supplementary Material, Table S2) and according to the following steps: (i) identification of titles and abstracts of records; (ii) removal of duplicates; (iii) screening and selection of titles and abstracts; (iv) evaluation of study eligibility through full-text studies; and (v) final inclusion in meta-analysis. Studies were screened and selected by 2 independent authors (S.D.A. and A.G.). If there was disagreement, the decision whether to include or exclude the study was made in consultation with a third senior author (F.F.).

The study protocol was registered and published online in PROSPERO (The International Prospective Register of Systematic Reviews; ID: CRD42022295917).

### Data extraction and database

Two authors (S.D.A. and A.G.) independently proceeded with data extraction, which was then reported in a standard table sheet database (Microsoft Office Excel 2016, Microsoft, Redmond, WA, USA). All studies included in the meta-analysis were listed by first author, country, study design and year of publication. The following patient baseline characteristics were collected: age, male gender, hypertension, diabetes, CVE, renal failure, chronic obstructive pulmonary disease, persistent/permanent atrial fibrillation, previous myocardial infarction, congestive heart failure, peripheral vascular disease, timing of surgery (urgency/emergency) and reoperation. The following peri- and postoperative variables were also collected: need for IABP, CVE, new onset of POAF, AKI, re-thoracotomy for bleeding/tamponade, PMV and early mortality.

### Primary and secondary endpoints

The primary endpoint was the cumulative long-term survival. The secondary endpoints were early mortality, defined as death occurred within 30 days or during the index admission, and the following postoperative variables: new onset of POAF, AKI, need for dialysis, PMV, CVE, IABP usage and re-thoracotomy for bleeding/tamponade.

### Statistical analysis

The pooled HR with 95% CI using the Mantzel–Haenszel method was calculated for survival and time-to-event analysis. The pooled effect size with odds ratio (OR) and 95% CI using the Mantzel–Haenszel method were calculated for the early mortality and for the other secondary endpoints. The random-effect model was preferred to account for variability among the studies. Forest plots were used to represent the effect sizes of primary and secondary endpoints.

Statistical heterogeneity was assessed according to the Galbraith plot and visual inspection of the forest plots. Heterogeneity was further evaluated with chi-squared and *I^2^* tests and defined as absent or low for *I^2^* ranging from 0% to 25%, moderate for *I^2^* ranging from 26% to 50% and high for *I^2^* above 50% [8].

Kaplan–Meier curve graphs were digitalized using GetData Graph Digitizer version 2.5.3 (http://getdata-graph-digitizer.com) and finally, the HR and the corresponding 95% CI were calculated by analysing the time-to-event outcomes according to the methods proposed by Tierney *et al.* [9]. Then, we reconstructed the original database of each article using the method described by Wei and Royston [10] and the survival curves of each study were reprocessed and visually compared with the original ones. Hence, all data were merged into a single database and survival curves and life tables were calculated. The log-rank test was used to evaluate differences between the 2 groups. A Cox proportional hazard model was used to calculate the HR between i-SAVR versus CABG+SAVR. We pre-planned to perform a landmark analysis in case of violation of the proportional hazard assumption test, indicated as a *P* < 0.05, to understand whether CABG added to SAVR affected long-term survival. In the event that the proportionality test is violated we expect to identify one or more cut-offs to analyse only those subjects who survived to the landmark time. Furthermore, a fully parametric model was used to obtain the time-dependent HRs (Royston–Parmar models) using a restricted cubic spline. Publication bias was assessed for each endpoint by creating the funnel plots using the trim and fill method and was analysed by means of Egger’s test [11] and visually expected for asymmetry. In case of non-proportional HR, we limited the HRs estimation and the publication bias only for survival at 5 years, as all included studies have at least 5-year follow-up. Sensitivity analysis was used to identify the possible influence of a single study by sequentially removing one study at a time, according to the leave-one-out method [12].

Continuous variables were reported as mean and standard deviation. Categorical variables were reported as number and percentages. A 2-tailed *P* < 0.05 indicated statistical significance except for tests on publication bias and heterogeneity, where a *P* < 0.1 indicates statistical significance. All comparisons were presented using the i-SAVR group as the reference. Statistical analyses were computed with ProMeta3 software (http://idostatistics.com/prometa3/), Stata/MP version 16.1 (Stata Corp, College Station, TX, USA) and Review Manager (RevMan5) Version 5.3 (The Cochrane Collaboration, 2012, The Nordic Cochrane Centre and Copenhagen, Denmark).

## RESULTS

The PRISMA Flow Chart of study selection process is shown in Supplementary Material, Fig. S1. A total of 730 titles and abstracts were identified, of which 23 were considered potentially relevant and retrieved as full text. After evaluating the full-text articles, 16 studies [2–6, 13–23] met the eligibility criteria and were included in the final analysis. Two authors (F.F. and A.G.) estimated the risk of bias assessment using the Newcastle–Ottawa Scale for observational studies (Supplementary Material, Fig. S2).

A total of 5382 patients were extracted from the selected articles and included in the analysis. The i-SAVR group consisted of 2568 patients (47.7%) and the CABG+SAVR group included 2814 patients (52.3%). Study and patient baseline characteristics are listed in Table 1.

**Table 1:** Baseline characteristics of the 15 studies included in the meta-analysis

Authors/country/year	Study design	i-SAVR	SAVR+CABG	Male gender		Hypertension		Diabetes		CVE	
				Overall (%)	By groups (%)	Overall (%)	By groups (%)	Overall (%)	By groups (%)	Overall (%)	By groups (%)
Brunvand/Norway/2002	Retrospective/single centre	42	52	36	i-SAVR: 21,4	N/A	N/A	N/A	N/A	N/A	N/A
					SAVR+CABG: 48						
Chiappini/Italy/2004	Retrospective/single centre	71	44	40.8	i-SAVR: N/A	44.8	i-SAVR: N/A	13.8	i-SAVR: N/A	5.2	i-SAVR: N/A
					SAVR+CABG: N/A		SAVR+CABG: N/A		SAVR+CABG: N/A		SAVR+CABG: N/A
Melby/USA/2007	Retrospective/single centre	105	140	53	i-SAVR: N/A	69	i-SAVR: N/A	18	i-SAVR: N/A	N/A	N/A
					SAVR+CABG: N/A		SAVR+CABG: N/A		SAVR+CABG: N/A		
Roberts/USA/2007	Retrospective/single centre	78	118	58	i-SAVR: N/A	N/A	N/A	N/A	N/A	N/A	N/A
					SAVR+CABG: N/A						
Huber/Swiss/2007	Retrospective/single centre	34	41	54.6	i-SAVR: 44.1	60	i-SAVR: 44	10.6	i-SAVR: 9	N/A	N/A
					SAVR+CABG: 63.4		SAVR+CABG: 73		SAVR+CABG: 13		
Likosky/USA/2009	Retrospective/multicentre	569	815	49.6	i-SAVR: 45	N/A	N/A	16.7	i-SAVR: N/A	N/A	N/A
					SAVR+CABG: 53				SAVR+CABG: N/A		
Maslow/USA/2010	Retrospective/single centre	145	116	45.6	i-SAVR: 51.7	78.2	i-SAVR: 78.6	22.6	i-SAVR: 17.9	N/A	N/A
					SAVR+CABG: 37.9		SAVR+CABG: 77.6		SAVR+CABG: 28.4		
Nikolaidis/UK/2011	Retrospective/single centre	161	184	N/A	N/A	N/A	N/A	6.9	i-SAVR: N/A	4.9	i-SAVR: N/A
									SAVR+CABG: N/A		SAVR+CABG: N/A
Kesavan/UK/2011	Retrospective/single centre	140	133	47	i-SAVR: N/A	N/A	N/A	11	i-SAVR: N/A	15	i-SAVR: N/A
					SAVR+CABG: N/A				SAVR+CABG: N/A		SAVR+CABG: N/A
Krane/Germany/2011	Retrospective/single centre	303	297	39.5	i-SAVR: 33.7	80.1	i-SAVR: N/A	20.6	i-SAVR: N/A	3.9	i-SAVR: 4.3
					SAVR+CABG: 45		SAVR+CABG: N/A		SAVR+CABG: N/A		SAVR+CABG: 3.4
Dell'Amore/Italy/2011	Retrospective/single centre	188	97	61.7	i-SAVR: 61.4%	77.9	i-SAVR: N/A	45.6	i-SAVR: N/A	N/A	N/A
					SAVR+CABG: 56		SAVR+CABG: N/A		SAVR+CABG: N/A		
Grau/USA/2014	Retrospective/single centre	87	102	55	i-SAVR: 61	N/A	N/A	28.9	i-SAVR: 30	7.5	i-SAVR: 7
					SAVR+CABG: 50				SAVR+CABG: 28		SAVR+CABG: 7
Wang/New Zealand/2016	Retrospective/single centre	93	104	64	i-SAVR: 60.2	57.3	i-SAVR: 51.6	11.2	i-SAVR: 7.5	5.6	i-SAVR: 8.4
					SAVR+CABG: 67		SAVR+CABG: 62.5		SAVR+CABG: 14.4		SAVR+CABG: 3.8
Kuo/Canada/2017	Retrospective/multicentre	170	208	58.1	i-SAVR: N/A	67.5	i-SAVR: N/A	19.6	i-SAVR: N/A	5.8	i-SAVR: N/A
					SAVR+CABG: N/A		SAVR+CABG: N/A		SAVR+CABG: N/A		SAVR+CABG: N/A
Ennker/Germany/2018	Retrospective/single centre	357	349	40.6	i-SAVR: 35	78.8	i-SAVR: 78.6	25.3	i-SAVR: 25.1	5.5	i-SAVR: 5.1
					SAVR+CABG: 48		SAVR+CABG: 79		SAVR+CABG: 25.5		SAVR+CABG: 5.9
Takagi/Japan/2020	Retrospective/single centre	18	11	17.2	i-SAVR: 28	86	i-SAVR: 89	20.7	i-SAVR: 22	20.7	i-SAVR: 11
					SAVR+CABG: 0		SAVR+CABG: 82		SAVR+CABG: 18		SAVR+CABG: 36

Authors/ country/year	Renal failure		COPD		Permanent AF		Previous AMI		Heart failure		PVD		Urgency		Redo surgery	
	Overall (%)	By groups (%)	Overall (%)	By groups (%)	Overall (%)	By groups (%)	Overall (%)	By groups (%)	Overall (%)	By groups (%)	Overall (%)	By groups (%)	Overall (%)	By groups (%)	Overall (%)	By groups (%)
Brunvand/Norway/2002	N/A	N/A	N/A	N/A	N/A	N/A	N/A	N/A	N/A	N/A	N/A	N/A	N/A	N/A	N/A	N/A
Chiappini/Italy/2004	N/A	N/A	19.8	i-SAVR: N/A	15.5	i-SAVR: N/A	12.1	i-SAVR: N/A	14.6	i-SAVR: N/A	N/A	N/A	14.7	i-SAVR: N/A	1.7	i-SAVR: N/A
				SAVR+CABG: N/A		SAVR+CABG: N/A		SAVR+CABG: N/A		SAVR+CABG: N/A				SAVR+CABG: N/A		SAVR+CABG: N/A
Melby/USA/2007	8	i-SAVR: N/A	12	i-SAVR: N/A	N/A	N/A	25	i-SAVR: N/A	N/A	N/A	13	i-SAVR: N/A	10	i-SAVR: N/A	10	i-SAVR: N/A
		SAVR+CABG: N/A		SAVR+ CABG: N/A				SAVR+CABG: N/A		N/A		SAVR+CABG: N/A		SAVR+CABG: N/A		SAVR+CABG: N/A
Roberts/USA/2007	N/A	N/A	N/A	N/A	N/A	N/A	N/A	N/A	N/A	N/A	N/A	N/A	N/A	N/A	N/A	N/A
Huber/Swiss/2007	21.3	i-SAVR: 21	18.6	i-SAVR: 21	25.3	i-SAVR: 29	10.6	i-SAVR: 6	N/A	N/A	12	i-SAVR: 12	N/A	N/A	N/A	N/A
		SAVR+CABG: 22		SAVR+CABG: 17		SAVR+CABG: 22		SAVR+CABG: 15				SAVR+CABG: 12				
Likosky/USA/2009	4.1	i-SAVR: N/A	6	i-SAVR: N/A	N/A	N/A	7.5	i-SAVR: 5.6	53.7	i-SAVR: 50	16.3	i-SAVR: 9.5	54.1	i-SAVR: 41.5	N/A	N/A
		SAVR+CABG: N/A		SAVR+ CABG: N/A				SAVR+CABG: 8.7		SAVR+CABG: 42.4		SAVR+CABG: 21		SAVR+CABG: 62.7		
Maslow/USA/2010	9.2	i-SAVR: 8.3	13.8	i-SAVR: 11	3.1	i-SAVR: 2.1	23	i-SAVR: 12.4	N/A	N/A	N/A	N/A	32.6	i-SAVR: 31.7	5.4	i-SAVR: 9.6
		SAVR+CABG: 10.3		SAVR+ CABG: 25.9		SAVR+CABG: 4.3		SAVR+CABG: 36.2						SAVR+CABG: 33.6		SAVR+CABG: 4.3
Nikolaidis/UK/2011	3.4	i-SAVR: N/A	11.6	i-SAVR: N/A	N/A	N/A	9	i-SAVR: N/A	7.5	i-SAVR: N/A	3.7	i-SAVR: N/A	52	i-SAVR: N/A	N/A	/
		SAVR+CABG: N/A		SAVR+CABG: N/A				SAVR+CABG: N/A		SAVR+CABG: N/A		SAVR+CABG: N/A		SAVR+CABG: N/A		/
Kesavan/UK/2011	4	i-SAVR: N/A	17	i-SAVR: N/A	N/A	N/A	15	i-SAVR: N/A	2	i-SAVR: N/A	9	i-SAVR: N/A	N/A	N/A	5	i-SAVR: N/A
		SAVR+CABG: N/A		SAVR+CABG: N/A				SAVR+CABG: N/A		SAVR+CABG: N/A		SAVR+CABG: N/A				SAVR+CABG: N/A
Krane/Germany/2011	N/A	N/A	N/A	N/A	24	i-SAVR: 28.1	9	i-SAVR: 4	N/A	/N/A	N/A	N/A	24.5	i-SAVR: 23.9	5.1	i-SAVR: N/A
						SAVR+CABG: 20		SAVR+CABG: 13.6						SAVR+CABG: 25.8		SAVR+CABG: N/A
Dell'Amore/Italy/2011	7.3	i-SAVR: N/A	14.7	i-SAVR: N/A	20.3	i-SAVR: N/A	21	i-SAVR: N/A	N/A	N/A	N/A	N/A	15	i-SAVR: N/A	1.4	i-SAVR: 2.1
		SAVR+CABG: N/A		SAVR+CABG: N/A		SAVR+CABG: N/A		SAVR+CABG: N/A						SAVR+CABG: N/A		SAVR+CABG: 1.1
Grau/USA/2014	N/A	N/A	16.6	i-SAVR: 14	N/A	N/A	N/A	N/A	N/A	N/A	10.6	i-SAVR: 9	N/A	N/A	13.2	i-SAVR: 19
				SAVR+CABG: 20								SAVR+CABG: 12				SAVR+CABG: 9
Wang/New Zealand/2016	1	i-SAVR: 1	23.3	i-SAVR: 36.8	28.5	i-SAVR: 27	31.5	i-SAVR: 14.1	19.8	i-SAVR: 25.8	13.7	i-SAVR: 9.7	56.3	i-SAVR: 49.5	13.2	i-SAVR: 4.3
		SAVR+CABG: 1.1		SAVR+CABG: 20.2		SAVR+CABG: 28		SAVR+CABG: 41.1		SAVR+CABG: 14.4		SAVR+CABG: 17.3		SAVR+CABG: 62.5		SAVR+CABG: 0
Kuo/Canada/2017	N/A	N/A	11.4	i-SAVR: N/A	18.8	i-SAVR: N/A	25.7	i-SAVR: N/A	N/A	/N/A	16.7	i-SAVR: N/A	36.2	i-SAVR: N/A	N/A	N/A
				SAVR+CABG: N/A		SAVR+CABG: N/A		SAVR+CABG: N/A				SAVR+CABG: N/A		SAVR+CABG: N/A		
Ennker/Germany/2018	N/A	N/A	N/A	N/A	19	i-SAVR: 20.5	N/A	N/A	N/A	N/A	9.9	i-SAVR: 8.6	N/A	N/A	8.8	i-SAVR: 9.8
						SAVR+CABG: 18.6						SAVR+CABG: 11.5				SAVR+CABG: 8.3
Takagi/Japan/2020	10	i-SAVR: 9	13.8	i-SAVR: 22	6.9	i-SAVR: 6	3.5	i-SAVR: 0	3.5	i-SAVR: 6	13.8	i-SAVR: 17	3.4	i-SAVR: 6	N/A	N/A
		SAVR+CABG: 11		SAVR+CABG: 0		SAVR+CABG: 9		SAVR+CABG: 9		SAVR+CABG: 0		SAVR+CABG: 9		SAVR+CABG: 0		

AF: atrial fibrillation; AMI: acute myocardial infarction; CABG: coronary artery bypass grafting; COPD: chronic obstructive pulmonary disease; CVE: cerebrovascular events; i-SAVR: isolated surgical aortic valve replacement; N/A: not available; PVD: peripheral vascular disease.

### Assessment of estimated reconstructed Kaplan–Meier curves

Visual comparison between the original reported Kaplan–Meier curves and the estimated reconstructed Kaplan–Meier curves was made and no differences were noted. When available, the reported HRs of selected studies were compared with the estimated HRs. No differences were reported among estimated and reported HRs (Supplementary Material, Fig. S3), and among the original and reconstructed survival curves, confirming an elevated accuracy of the reconstructed data.

### Primary endpoint: long-term mortality

All studies included in the meta-analysis reported the long-term mortality and the related Kaplan–Meier curves comparison between i-SAVR and CABG+SAVR. Across the included studies, the reported mean follow-up ranged between 2.4 [16] and 6.6 years [18] with a weighted mean follow-up of 5.1 years. The longest follow-up was 15 years [22]. All included studies reported at least 5-year follow-up.

**Figure 1:**
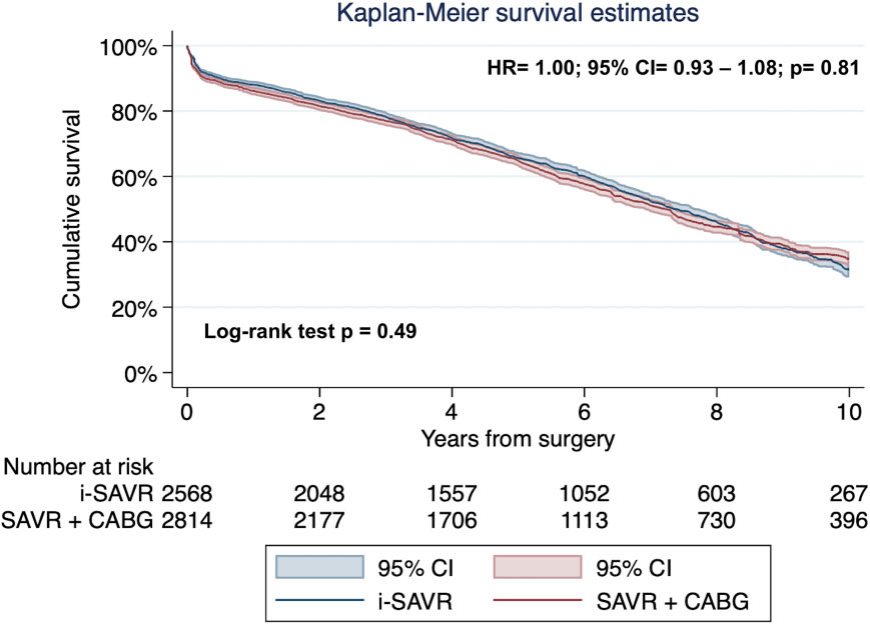
Pooled reconstructed Kaplan–Meier survival curves for long-term survival. Non-difference was reported between the 2 interventions. CABG: coronary artery bypass grafting; CI: confidence interval; HR: hazard ratio; i-SAVR: isolated surgical aortic valve replacement.

**Figure 2:**
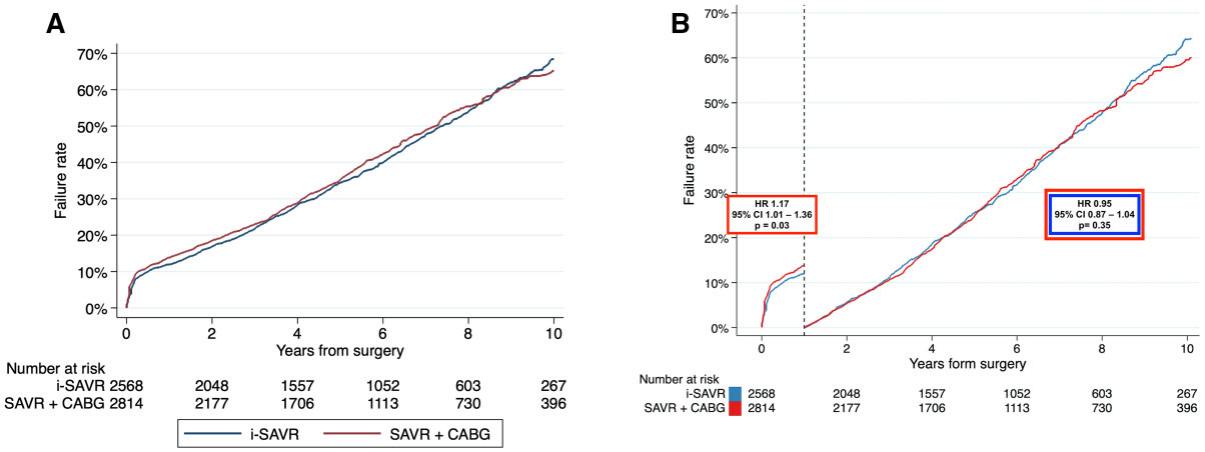
(**A**) Kaplan–Meier of failure function of the pooled all-cause mortality. (**B**) Landmark analysis of all-cause mortality. CABG: coronary artery bypass grafting; HR: hazard ratio; i-SAVR: isolated surgical aortic valve replacement.

The pooled Kaplan–Meier curves are illustrated in Fig. 1. No difference was revealed between the 2 groups (log-rank test, *P* = 0.49). The Cox proportional hazard model revealed no difference between the 2 populations (HR = 1.00; 95% CI = 0.93–1.08; *P* = 0.81). Survival at 5 and 10 years were 65.3% (95% CI = 63.3-67.2%) and 32.1% (95% CI = 29.6–34.7%), respectively, in i-SAVR population; in CABG+SAVR patients, survival at 5 and 10 years were 64% (95% CI = 62.1–65.8%) and 34.8% (95% CI = 32.6–37.7%), respectively. The test for the proportional hazard assumption was violated (*P* = 0.004). Therefore, we proceeded with the landmark and the time-dependent HR analyses, applying the cut-off of 1 year, according to the visual inspection of scaled Schoenfeld residuals and the Kaplan–Meier curves. Within the first year after surgery, CABG+SAVR was associated with a significant higher incidence of mortality (HR = 1.17; 95% CI = 1.01–1.36; *P* = 0.03), while from 1- to 10-year follow-up, no significant differences were observed between the 2 interventions (HR = 0.95; 95% CI = 0.87–1.04; *P* = 0.35; Fig. 2A and B).

**Figure 3:**
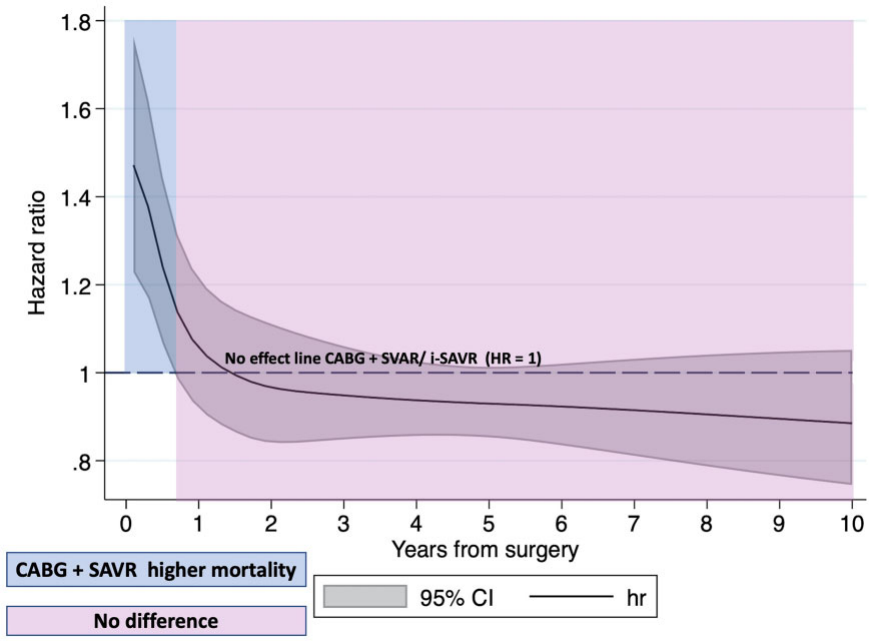
Hazard ratio trend over time for all-cause mortality estimated by fully parametric survival models. CABG: coronary artery bypass grafting; CI: confidence interval; HR: hazard ratio; i-SAVR: isolated surgical aortic valve replacement.

The analysis of the HR trend over time of i-SAVR versus CABG+SAVR by fully parametric survival models confirmed the results of the landmark analysis (Fig. 3).

Because we demonstrated the non-proportionality of HRs over time, we assessed the publication bias and study heterogeneity at a well-defined time point, corresponding to 5 years of follow-up, as all included studies reported at least 5 years of follow-up. The pooled analysis of long-term survival revealed no difference (HR = 0.96; 95% CI = 0.86–1.08; *P* = 0.51) and evidence of moderate heterogeneity (*I*^2^ = 29%; Supplementary Material, Fig. S4). No evidence of publication bias was found assessed by the Egger’s test (*P* = 0.77) or visual inspection of the funnel plot (Supplementary Material, Fig. S5). Heterogeneity was assessed across the studies according to the Galbraith plot (Supplementary Material, Fig. S6).

An additional sensitivity analysis was performed using effect estimates based on logHR (Supplementary Material, Fig. S7). No difference was found between the 2 groups at time point of 5-year follow-up.

### Secondary outcomes

**Figure 4:**
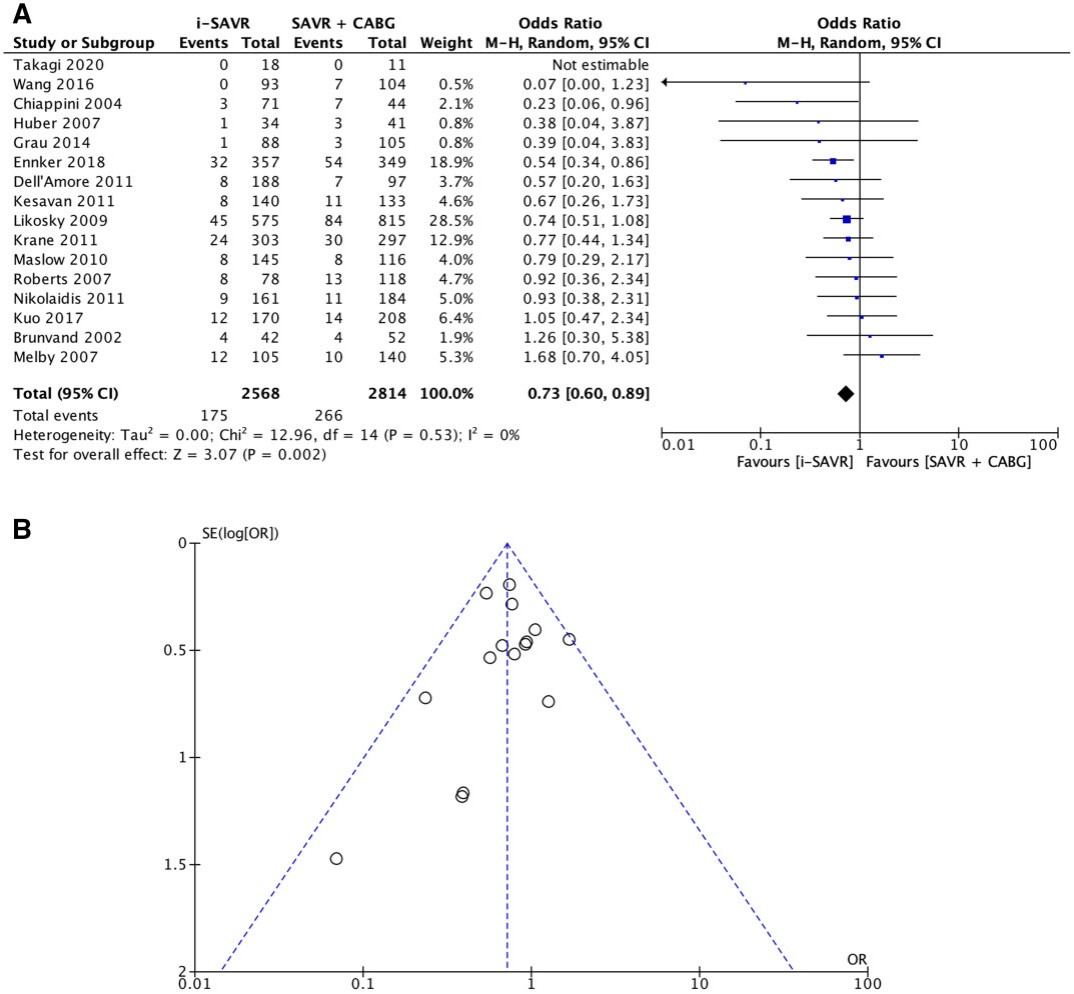
(**A**) Forest plot for early mortality. Isolated aortic valve replacement (i-SAVR) was associated with lower early mortality compared to coronary artery bypass grafting (CABG)+SAVR. *I*^2^, 0.87% indicates no evidence of heterogeneity. CABG: coronary artery bypass grafting; CI: confidence interval; i-SAVR: isolated surgical aortic valve replacement; OR: odd ratio; Sig: *P*-value; W: weight. (**B**) Funnel plot to assess publication bias. No publication bias was reported related to early mortality.

All included studies reported data on early mortality. The OR revealed a significant difference, favouring i-SAVR versus CABG+SAVR (OR = 0.73; 95% CI = 0.60–0.89; *P* = 0.002) with no significant heterogeneity (*I*^2^ = 0%; Fig. 4A) across the studies. No evidence of publication bias was found assessed by the Egger’s test (*P* = 0.29) or visual inspection of the funnel plot (Fig. 4B).

The leave-one-out analysis did not identify any influential studies on the pooled data (Supplementary Material, Fig. S8).

Five studies presented data on new-onset POAF [3, 4, 15–17]. The OR did not show significant difference between the 2 groups (OR = 0.87; 95% CI = 0.68–1.12; *P* = 0.28) with moderate heterogeneity (*I*^2^ = 42%; Supplementary Material, Fig. S9).

Five studies presented data on postoperative AKI [3, 4, 17, 19, 20]. The OR did not reveal any difference between the 2 groups (OR = 0.68; 95% CI = 0.38–1.23; *P* = 0.20) with significative heterogeneity (*I*^2^ = 61%; Supplementary Material, Fig. S10).

Four studies reported data on PMV [3, 4, 17, 20]. The OR revealed a significant difference, favouring i-SAVR compared to CABG+SVAR (OR = 0.55; 95% CI = 0.39–0.77; *P* < 0.001) without evidence of heterogeneity (*I*^2^ = 0%; Supplementary Material, Fig. S11) across the studies.

Nine studies presented data on postoperative CVE [3, 4, 15–17, 19, 20, 22, 23]. There was no difference between the 2 groups (OR = 0.92; 95% CI = 0.61–1.39; *P* = 0.69) and no heterogeneity was revealed across the studies (*I*^2^ = 0%; Supplementary Material, Fig. S12).

Data on postoperative IABP usage were reported in 4 studies [3, 4, 15–19]. No difference was revealed (OR = 0.58; 95% CI = 0.24–1.40; *P* = 0.22) between the 2 groups and no considerable heterogeneity (*I*^2^ = 19%; Supplementary Material, Fig. S13) across the studies.

Seven studies reported data on re-thoracotomy for postoperative bleeding [3, 4, 6, 15–17, 20, 23]. The OR did not reveal significant difference between the 2 groups (OR = 0.84; 95% CI = 0.57–1.23; *P* = 0.37) with no relevant heterogeneity (*I*^2^ = 21%; Supplementary Material, Fig. S14) across the studies.

No evidence of publication bias was found assessed by the Egger’s tests and visual inspection of the funnel plots (Supplementary Material, Figs. S15–S20).

## DISCUSSION

In the last 50 years, there has been a relevant interest for the role of cardiac operations in aged patients older than 80 years. This upward trend is based firstly, on the increase in the elderly population due to longer life expectancy, secondly on the improved general condition of the aged population, and finally on the availability of safer surgical techniques that allow intervention even on higher-risk patients.

In this updated meta-analysis, no unfavourable impact of CABG in combination with SAVR was reported on long-term survival compared to i-SAVR. The favourable outcome of i-SAVR compared with CABG+SAVR in the early term is reflected in a clear reduction in the operative mortality rate, while the impact of CABG in the long-term follow-up is still debated. Due to the violation of the proportional hazard assumption test, we applied the landmark analysis with a 1-year cut-off point, to observe the impact of CABG added to SAVR over time. The comparable long-term survival between the 2 interventions after 1 year from surgery can support the rationale that CAD associated with aortic valve disease, although an additional risk factor for early outcome, probably does not increase the long-term mortality when treated with CABG. Some authors reported a long-term benefit of patients with concomitant CAD and AS who underwent CABG+SAVR compared to those patients who did not receive CABG procedure at the time of SAVR [24]. Furthermore, the relief of the AS and the addiction of coronary revascularization would increase the coronary flow reserve and would provide the reversing remodelling as in patients with isolated AS who underwent i-SAVR. These factors contribute for the regression of left ventricle hypertrophy and increased coronary microcirculation which are determinant for the long-term survival [25].

To the best of our knowledge, this is the first reconstructed time-to-event and time-varying effect meta-analysis regarding the long-term impact of CABG added to SAVR in octogenarian patients. The main findings were: (i) the long-term survival was comparable between the 2 interventions; (ii) the in-hospital mortality would appear to be higher with the combination of CABG and SVAR; and (iii) regarding the postoperative complications analysed, the i-SVAR is associated with a reduced rate of PMV.

Over the last decade, the transcatheter approach for elderly patients with AS has been increasing, even in low-risk patients, with encouraging early and mid-term results compared to conventional surgery. However, the problem remains for that large group of patients with concomitant AS and CAD, for whom the transcatheter approach with PCI and TAVI has not yet yielded encouraging results. In a recent meta-analysis including 3 multicentre studies, no differences were found between the TAVI plus PCI and the CABG plus SAVR [26]. Specifically, 30-day mortality, stroke, myocardial infarction and 2-year mortality were similar in both groups. Although the authors observed no relevant differences between the 2 groups, in terms on early and late mortality, the high heterogeneity and the small number of selected studies, and the high heterogeneity of the revascularization strategies in the TAVI plus PCI group, ranging from simultaneous to staged approach, prevent drawing conclusions. Data from the randomized SURTAVI trial, comparing the TAVI plus PCI strategy with the SAVR plus CABG intervention, reported comparable 30-day mortality, disabling stroke and myocardial infarction, while the rate of postoperative major vascular complications, and pacemaker implant were statistically higher in transcatheter group, instead rate of acute kidney injuries and POAF were higher in surgical group [27]. Mortality and disabling stroke were comparable in both groups 2 years after the assigned treatment. However, the study randomized patients with SYNTAX-score <22, therefore with a favourable CAD for PCI.

In this meta-analysis, it is interesting to highlight that the negative impact that CABG added to SAVR would have during the postoperative period, might became non-significant over time, as demonstrated by the same survival probability reported in the 2 groups at 10 years after surgery. The 5- and 10-year survivals of ∼66% and 34%, respectively, represent satisfactory results that are important in affirming both the validity and safety of the conventional surgical approach and also that associated CBAG does not have an adverse long-term impact. We furtherly sought to investigate and explain the impact of CABG added to i-SAVR on common postoperative complications such as new-onset POAF, AKI, CVE, need for IABP, re-thoracotomy for bleeding/tamponade and PMV. Within these complications, only PMV had a significant increase in CABG+SVAR compared with i-SAVR, whereas the other complications had comparative incidences between the 2 groups. However, because these variables were not reported in each study, we did not have robust data to draw relevant and scientific soundness conclusions.

### Limitations

The meta-analysis shares the limitation of meta-analyses of retrospective observational studies that can be affected from a risk of treatment allocation bias and from unmeasured confounders. In addition, it was not possible to extrapolate the incidence of incomplete myocardial revascularization or the presence of moderated CAD or small coronary arteries, which were not addressed with CABG. Furthermore, the functional assessment of CAD by fractional flow reserve study was not reported. Therefore, in these scenarios, the impact of CABG+SAVR was not analysed.

It is noteworthiness to consider the inability to fully investigate the impact of CABG in postoperative complications, because in some of the included studies these were not fully reported. However, the primary endpoint of the meta-analysis was to investigate the impact of CABG added to SAVR on long-term survival. We must also acknowledge that none of the studies included in the meta-analysis reported results after balancing the covariates with propensity score matching. Lack of adjustment may obviously lead to a risk of bias that we are unable to control for.

Another limitation is that not all the included studies have reported the type of bioprosthesis in terms of profile and structure and therefore it was not possible to add further analysis based on this issue.

Finally, it was not possible to extrapolate from each study the criteria to select patients for either conventional surgery or TAVI; therefore, these results are likely representative of an aged population who were fit for conventional SAVR.

## CONCLUSIONS

In this reconstructed time-to-event meta-analysis of studies comparing octogenarians undergoing either i-SAVR or CABG+SAVR, late survival did not differ significantly between the 2 interventions. Interestingly, CABG+SAVR is associated with both higher in-hospital mortality and within 1 year after surgery, whereas in the long-term follow-up this difference is reduced and becomes non-significant.

For octogenarian patients who need treatment for AS associated with CAD, it is useful to consider the favourable impact of CABG on long-term survival.

## SUPPLEMENTARY MATERIAL

Supplementary material is available at *ICVTS* online.

## Supplementary Material

ivac164_Supplementary_Data

## ABBREVIATIONS

AKI: Acute kidney injury
AS: Aortic stenosis
CABG: Coronary artery bypass grafting
CAD: Coronary artery disease
CI: Confidence interval
CVE: Cerebrovascular events
HR: Hazard ratio
IABP: Intra-aortic balloon pump
i-SAVR: Isolated surgical aortic valve replacement
OR: Odds ratio
PCI: Percutaneous coronary intervention
PMV: Prolonged mechanical ventilation
POAF: Postoperative atrial fibrillation
TAVI: Transcatheter aortic valve implantation
