# Supplementary material for: The impact of coronary artery bypass grafting added to aortic valve replacement on long-term outcomes in octogenarian patients: a reconstructed time-to-event meta-analysis
Source: Interact Cardiovasc Thorac Surg. 2022 Jun 20;35(2):ivac164. doi: 10.1093/icvts/ivac164 (PMC9272063; doi:10.1093/icvts/ivac164)
Supplement: ivac164_Supplementary_Data [file ivac164_supplementary_data.docx]

**The impact of coronary artery bypass grafting added to aortic valve replacement on long-term outcomes in octogenarian patients. A reconstructed time-to-event meta-analysis.**

**Alan Gallingani^2^, Stefano D’Alessandro^3^, Gurmeet Singh^4^, Daniel Hernandez-Vaquero^5^, Mevlüt Çelik^6^, Evelina Ceccato^7^, and Francesco Nicolini^1,2^ and Francesco Formica^1,2^.**

***^SUPPLEMENTAL MATERIALS^***

**Figure S1.** Study selection on the basis of the Preferred Reporting Items for Systematic Reviews and Meta-Analysis (PRISMA) Flow Diagram. A total of 835 title and abstracts were evaluated; 23 articles were assessed for eligibility; finally, 16 studies met all the inclusion criteria and were included in the analysis.

**Figure S2.** The Newcastle-Ottawa Scale for observational studies to assess the level of quality of studies included in the analysis.

**Figure S3.** Quality assessment of estimated and reported hazard ratios for long-term survival.

**Figure S4.** Forest plot for overall long-term survival at 5-years follow-up. No difference was observed between isolated aortic valve replacement (i-SAVR) and SAVR + coronary artery bypass grafting (CABG). I^2^, 29% indicates moderate heterogeneity.

**Figure S5.** Funnel plot to assess publication bias for overall long-term survival at 5-years follow-up.

**Figure S6.** Galbraith plot to assess heterogeneity across the study at 5-years follow-up.

**Figure S7.** Additional sensitivity analysis using effect estimates based on logHR.

**Figure S8**. The sensitivity analysis according to the leave-one-out method to identify any influential studies on the pooled data for early mortality.

**Figure S9**. Forest plot for new onset postoperative atrial fibrillation. No difference was observed between isolated aortic valve replacement (i-SAVR) and SAVR + coronary artery bypass grafting (CABG). I^2^, 42% indicates moderate heterogeneity.

**Figure S10**. Forest plot for postoperative acute renal failure. No difference was observed between isolated aortic valve replacement (i-SAVR) and SAVR + coronary artery bypass grafting (CABG). I^2^, 61% indicates significative heterogeneity.

**Figure S11**. Forest plot for prolonged mechanical ventilation. No difference was observed between isolated aortic valve replacement (i-SAVR) and SAVR + coronary artery bypass grafting (CABG). I^2^, 0% indicates no evidence of heterogeneity.

**Figure S12**. Forest plot for postoperative cerebrovascular events. No difference was observed between isolated aortic valve replacement (i-SAVR) and SAVR + coronary artery bypass grafting (CABG). I^2^, 0% indicates no evidence of heterogeneity.

**Figure S13**. Forest plot for postoperative intra-aortic balloon pump. No difference was observed between isolated aortic valve replacement (i-SAVR) and SAVR + coronary artery bypass grafting (CABG). I^2^, 19% indicates low heterogeneity.

**Figure S14**. Forest plot for re-thoracotomy for bleeding/tamponade. No difference was observed between isolated aortic valve replacement (i-SAVR) and SAVR + coronary artery bypass grafting (CABG). I^2^, 21 % indicates low heterogeneity.

**Figure S15**. Funnel plot to assess publication bias. No publication bias was reported related to postoperative atrial fibrillation.

**Figure S16**. Funnel plot to assess publication bias. No publication bias was reported related to postoperative acute renal failure.

**Figure S17**. Funnel plot to assess publication bias. No publication bias was reported related to prolonged mechanical ventilation.

**Figure S18**. Funnel plot to assess publication bias. No publication bias was reported related to postoperative cerebrovascular events.

**Figure S19**. Funnel plot to assess publication bias. No publication bias was reported related to postoperative intra-aortic balloon pump.

**Figure S20**. Funnel plot to assess publication bias. No publication bias was reported related to re-thoracotomy for bleeding/tamponade.

**Figure S1.** Study selection on the basis of the Preferred Reporting Items for Systematic Reviews and Meta-Analysis (PRISMA) Flow Diagram. A total of 835 title and abstracts were evaluated; 23 articles were assessed for eligibility; finally, 16 studies met all the inclusion criteria and were included in the analysis.


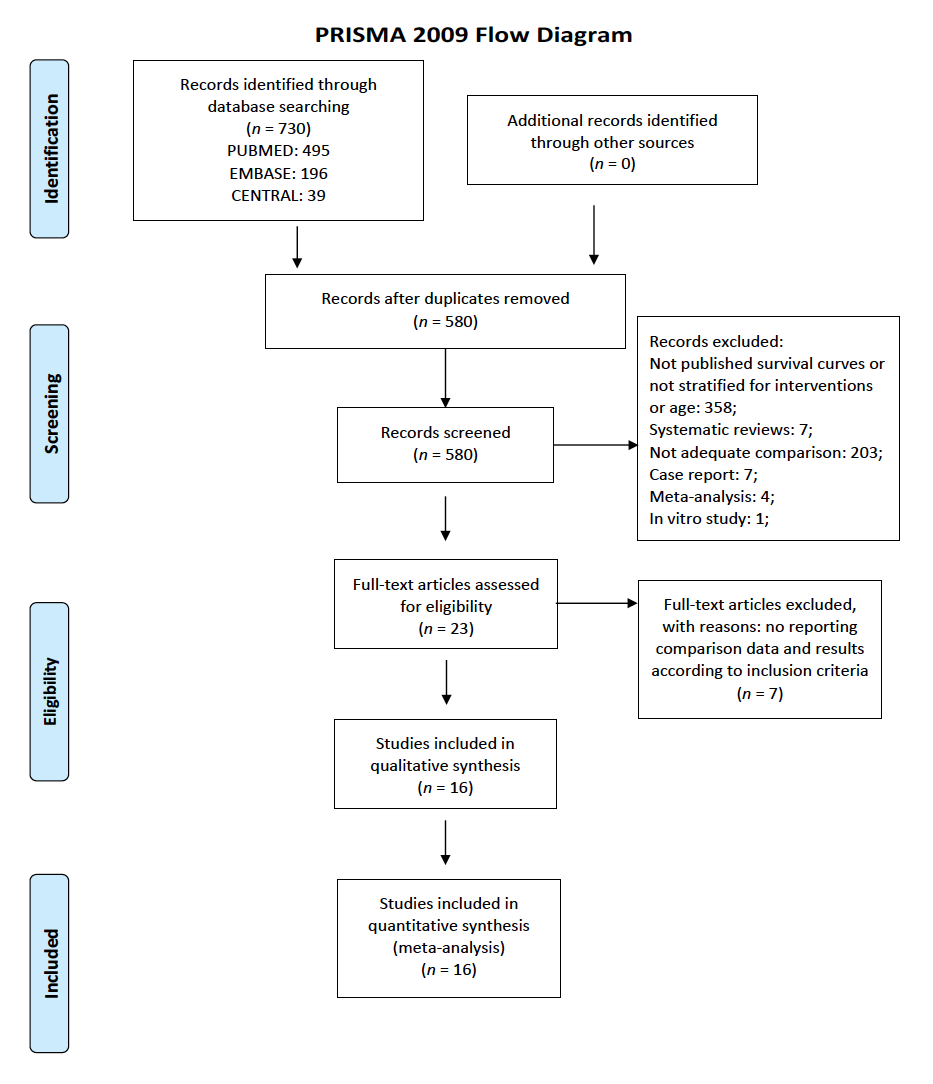


**Figure S2.** The Newcastle-Ottawa Scale for observational studies to assess the level of quality of studies included in the analysis.


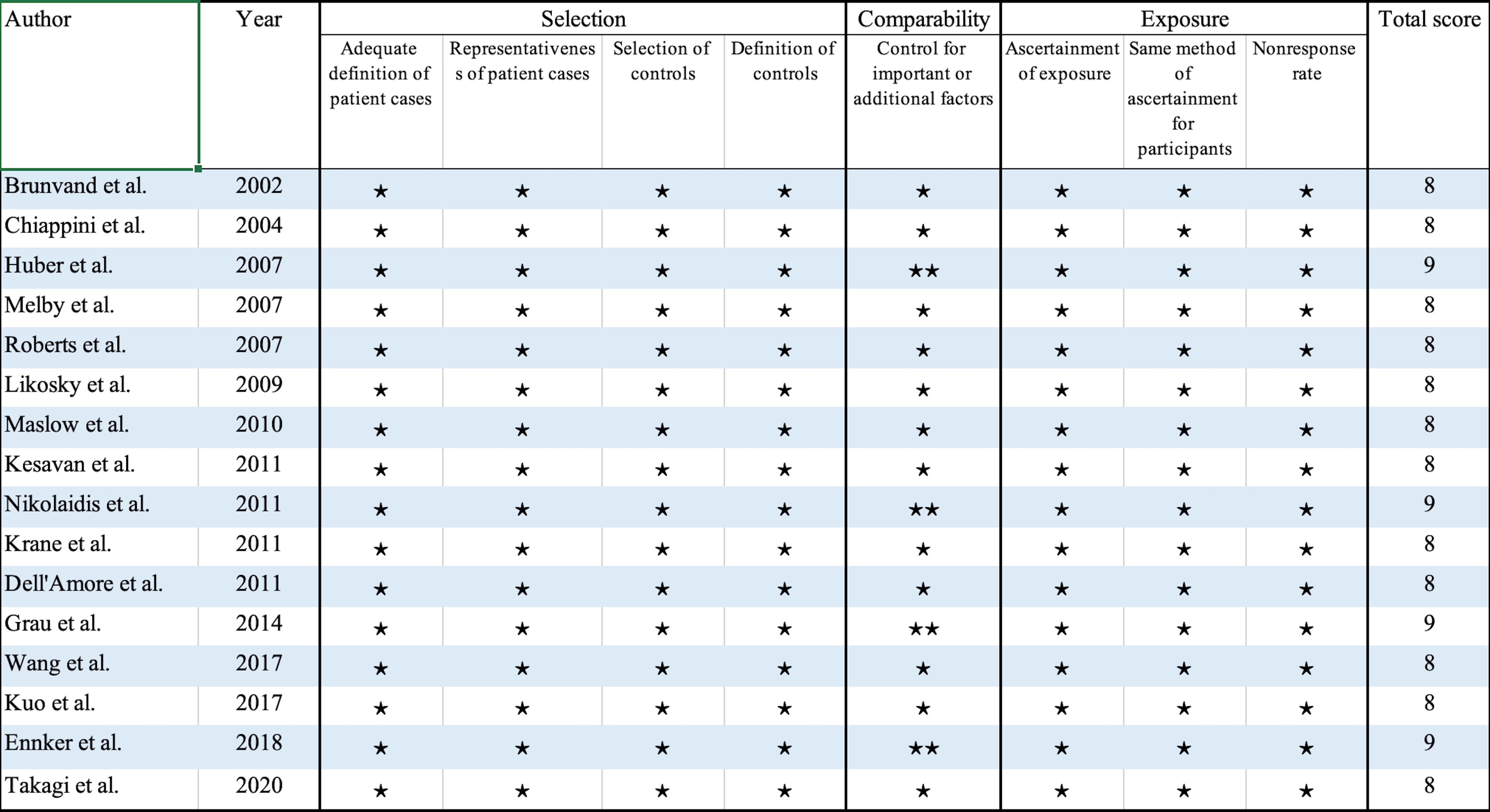


**Figure S3:** Quality assessment of estimated and reported hazard ratios for long-term survival.


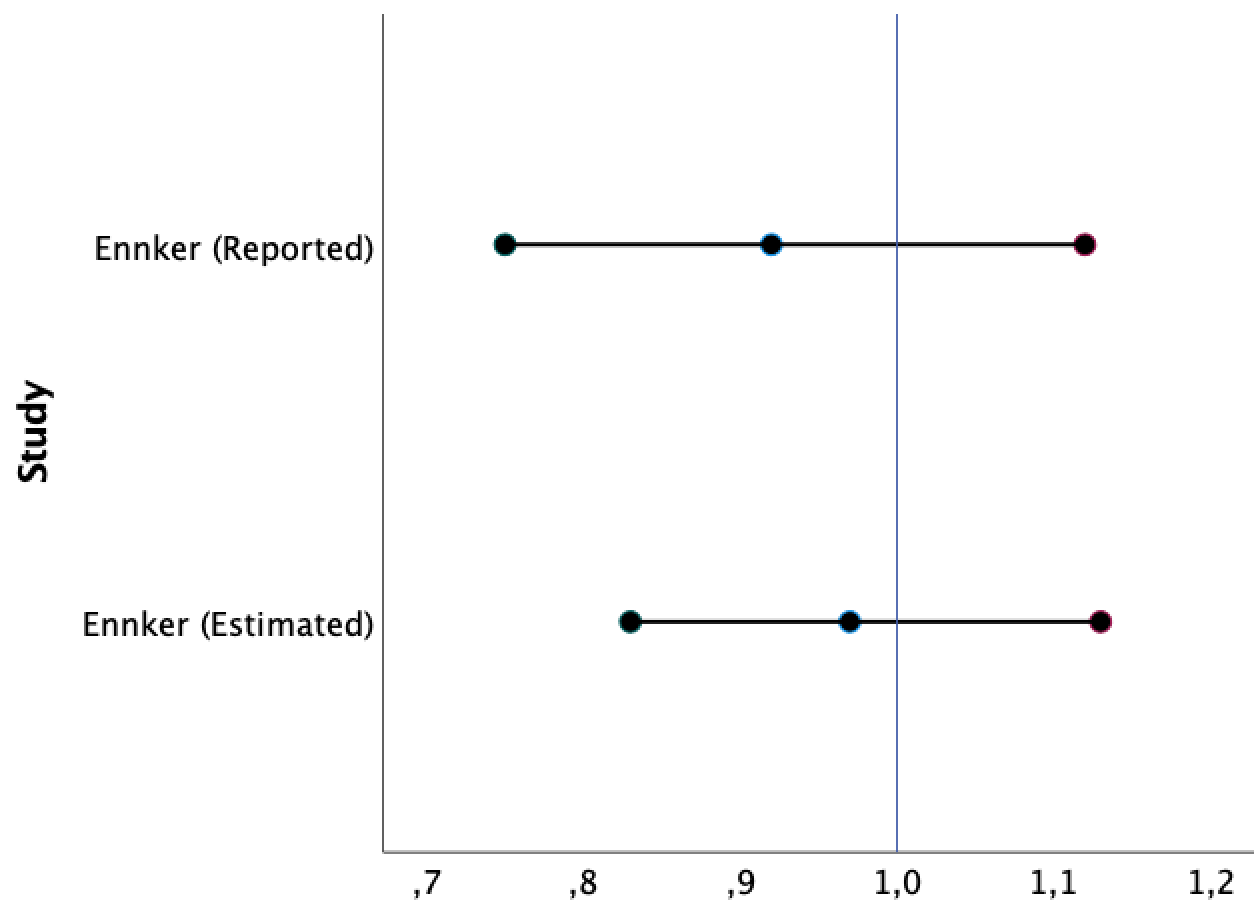

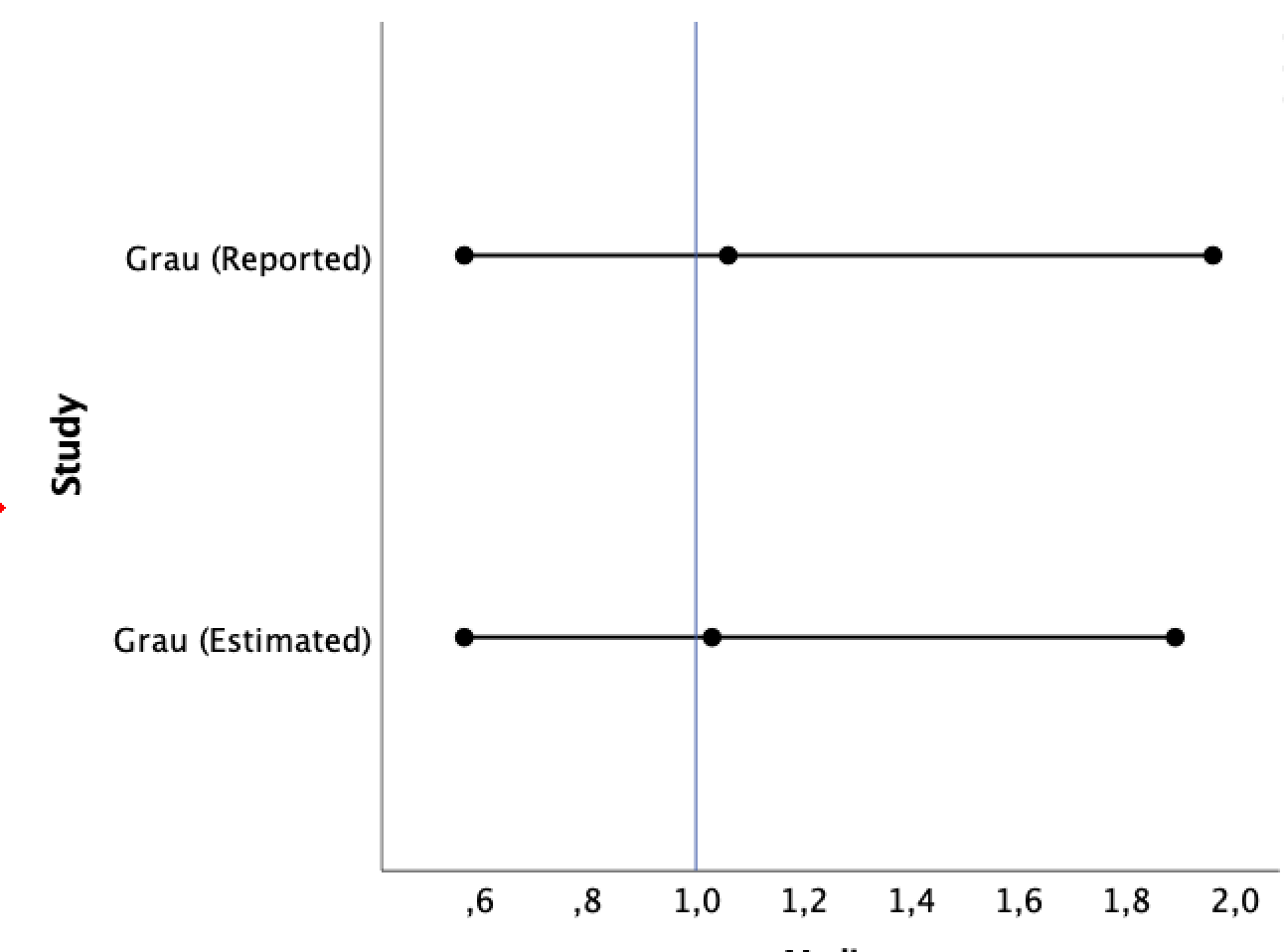


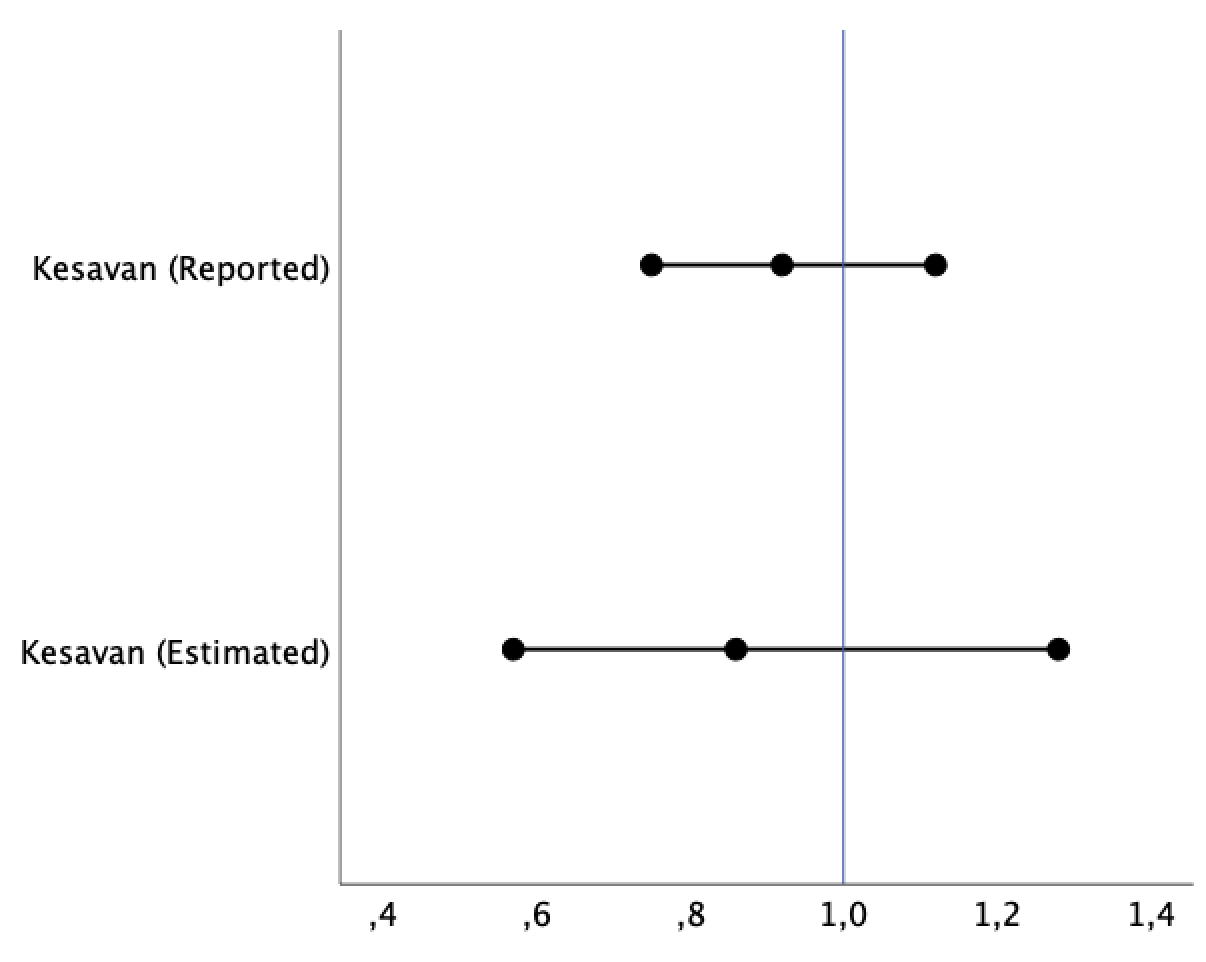

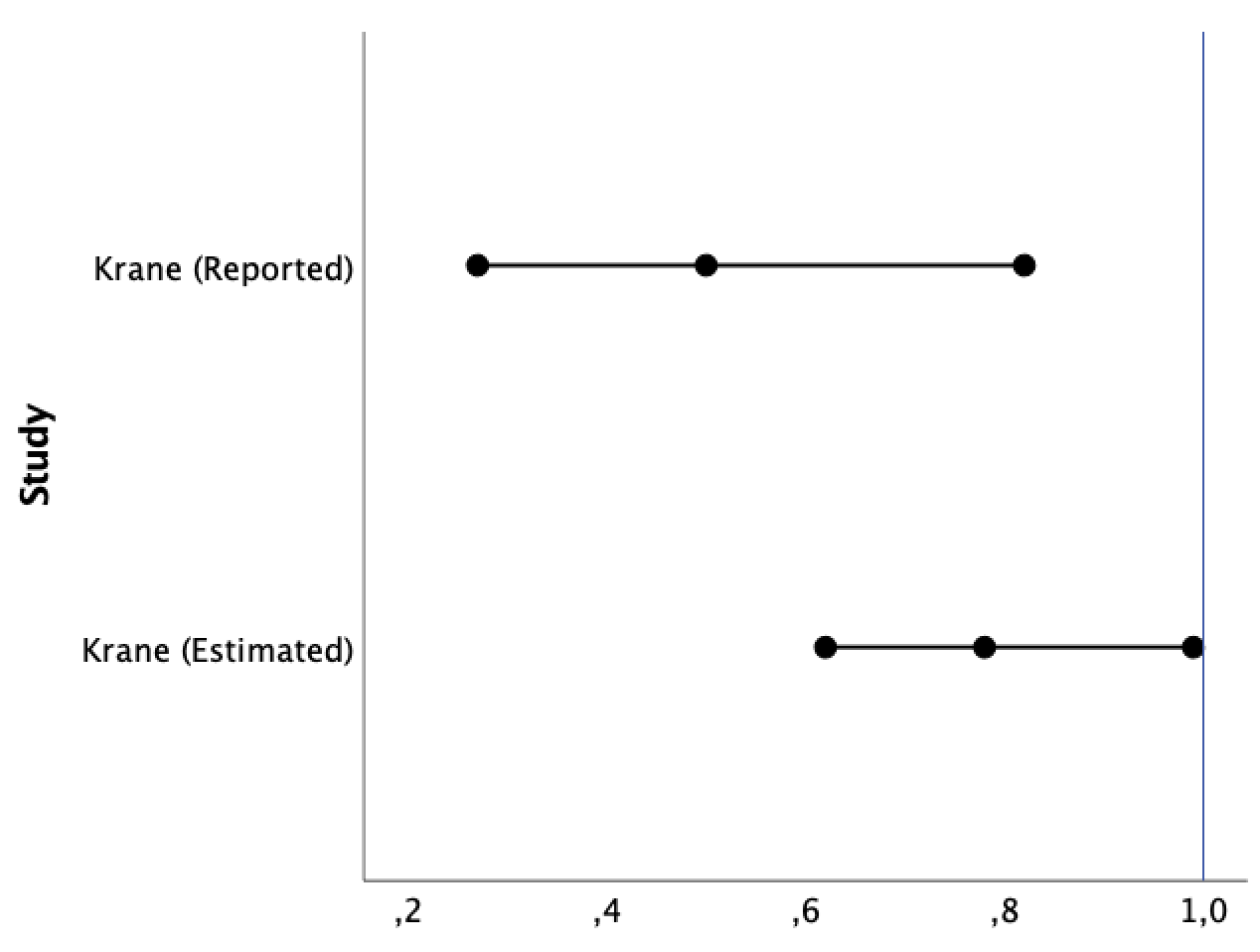


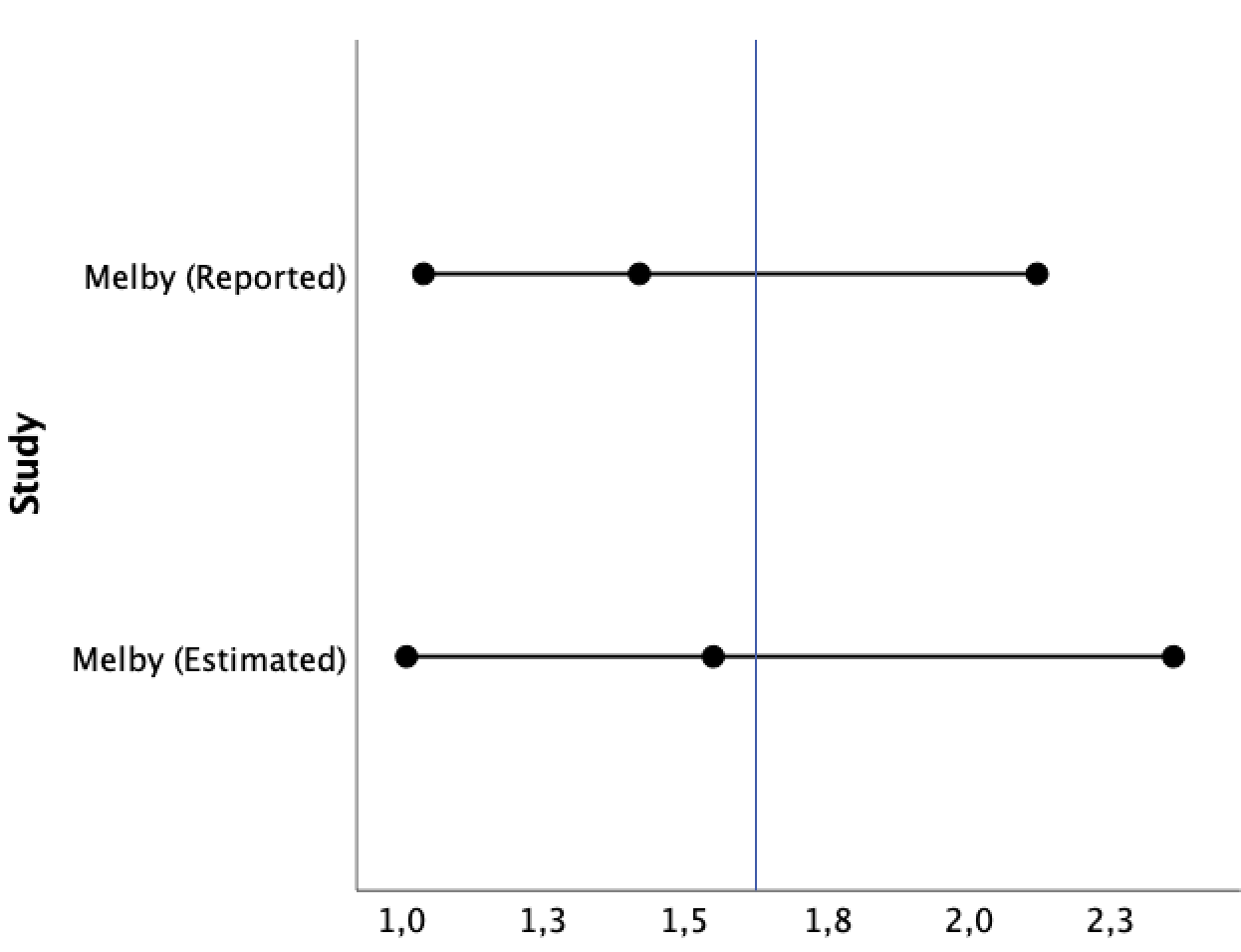

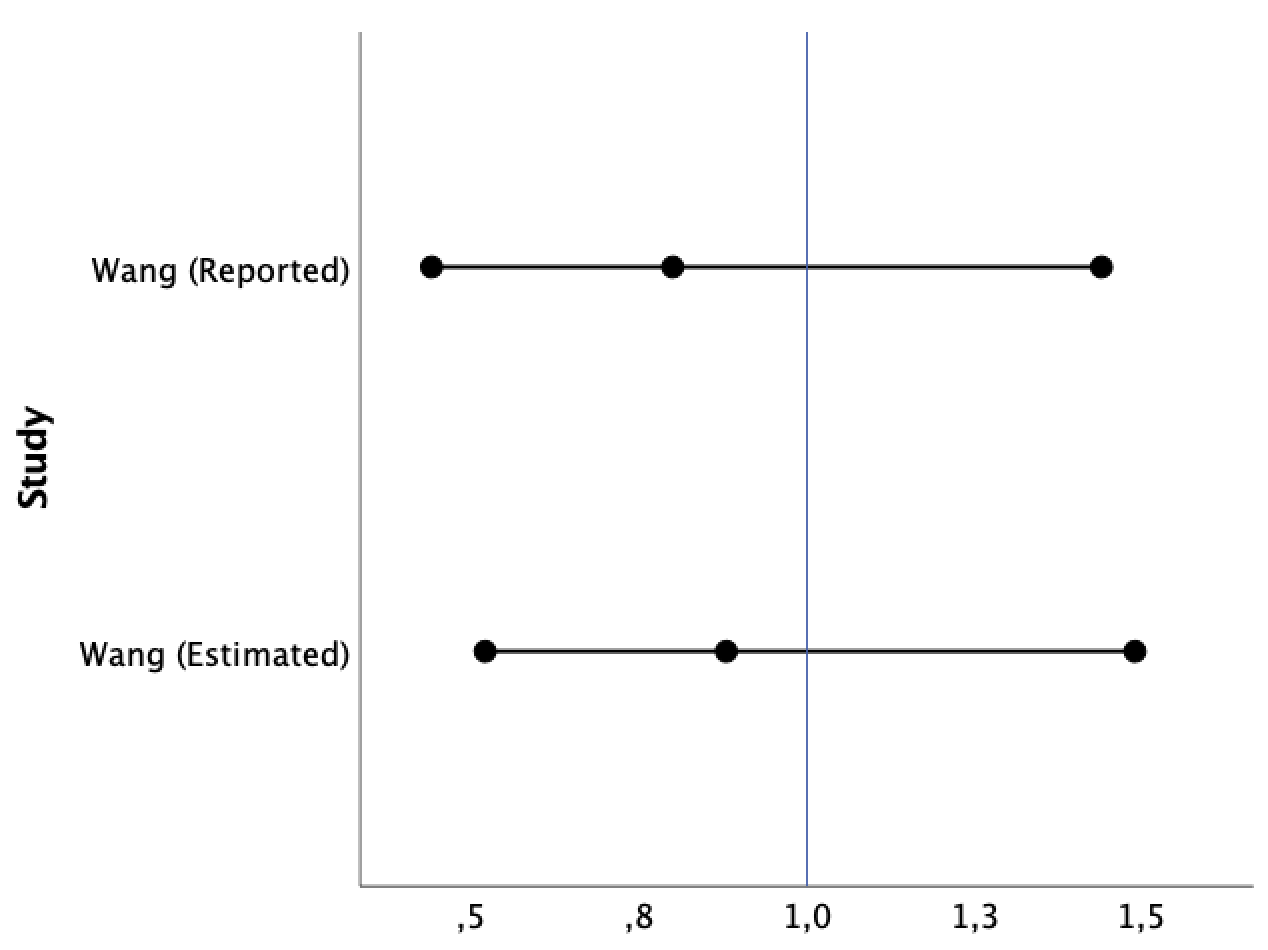


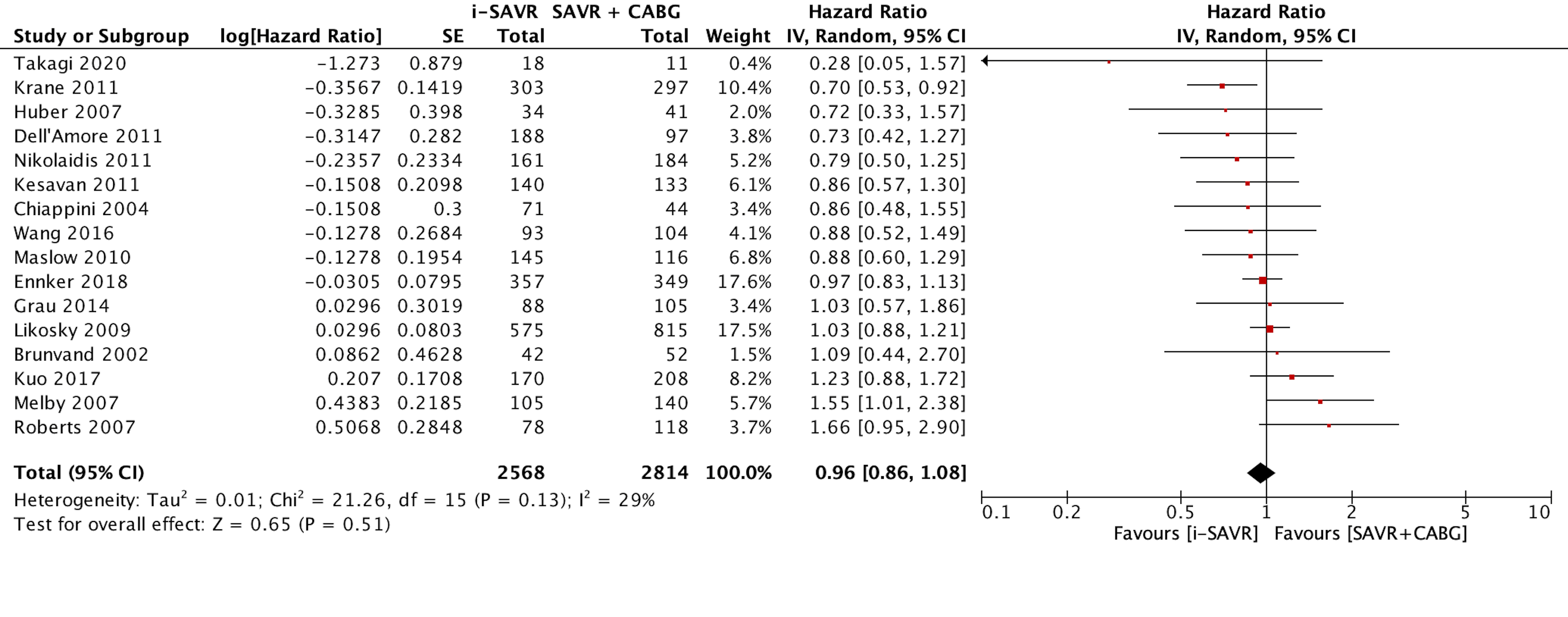


**Figure S4.** Forest plot for overall long-term survival at 5-years follow-up. No difference was observed between isolated aortic valve replacement (i-SAVR) and SAVR + coronary artery bypass grafting (CABG). I^2^, 29% indicates moderate heterogeneity.

*HR, hazard ratio; CI, confidence interval; W, weight; Sig, P value.*

*
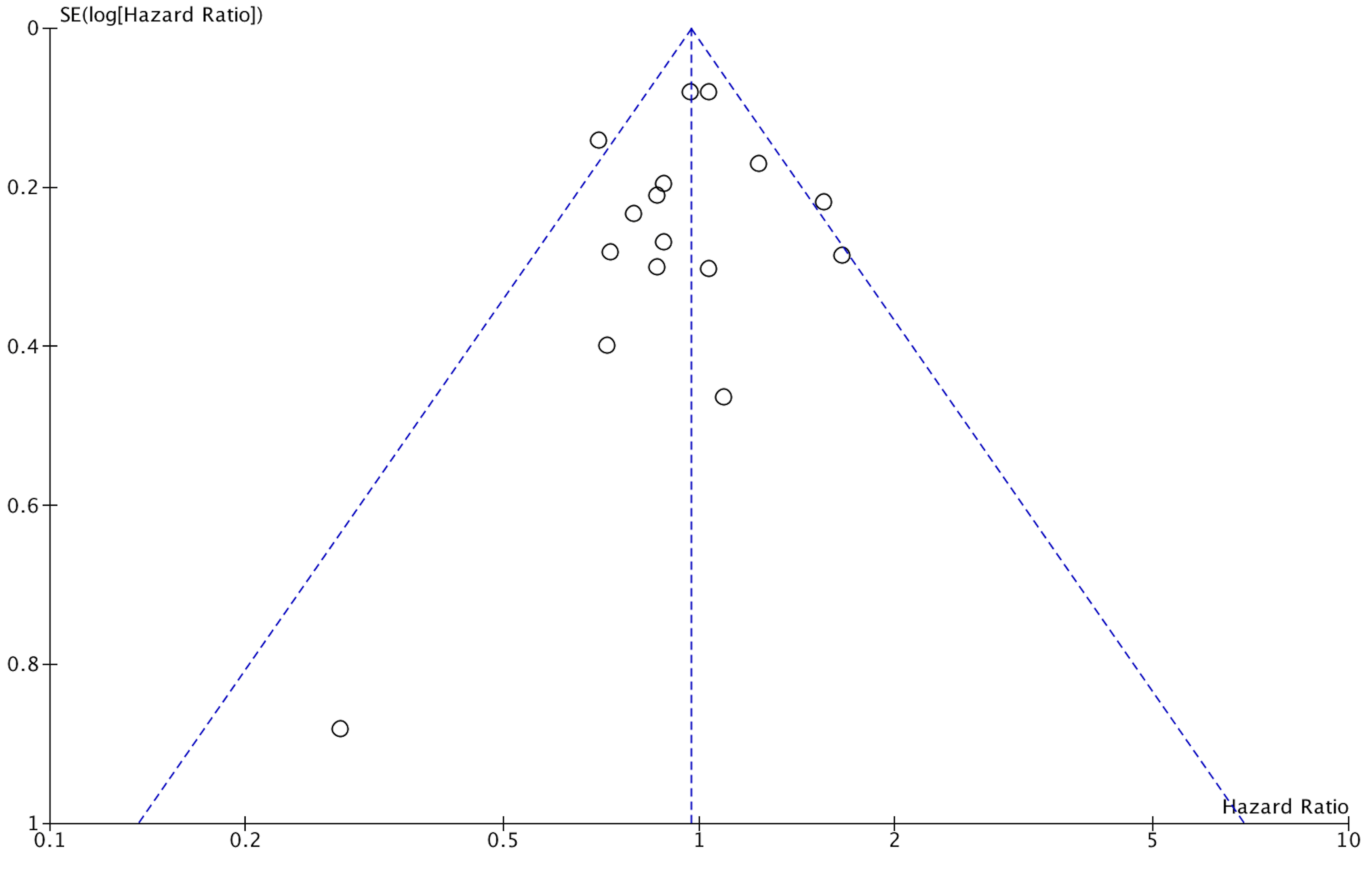
*

**Figure S5.** Funnel plot to assess publication bias for overall long-term survival at 5-years follow-up. No publication bias was reported related to postoperative atrial fibrillation. Egger’s test, p = 0.38.


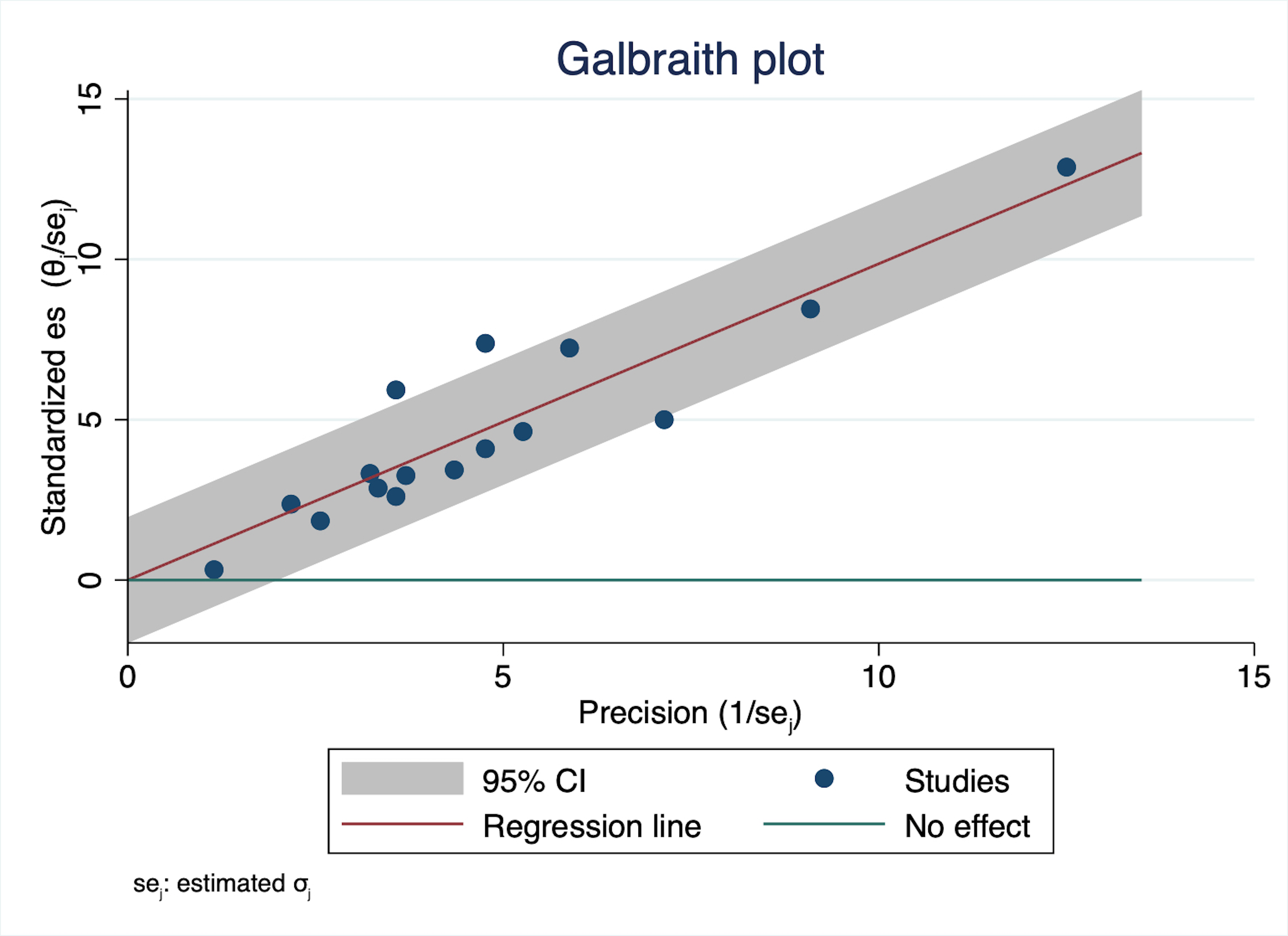


**Figure S6.** Galbraith plot to assess heterogeneity across the study at 5-years follow-up. All studies reported at least 5-years follow-up.


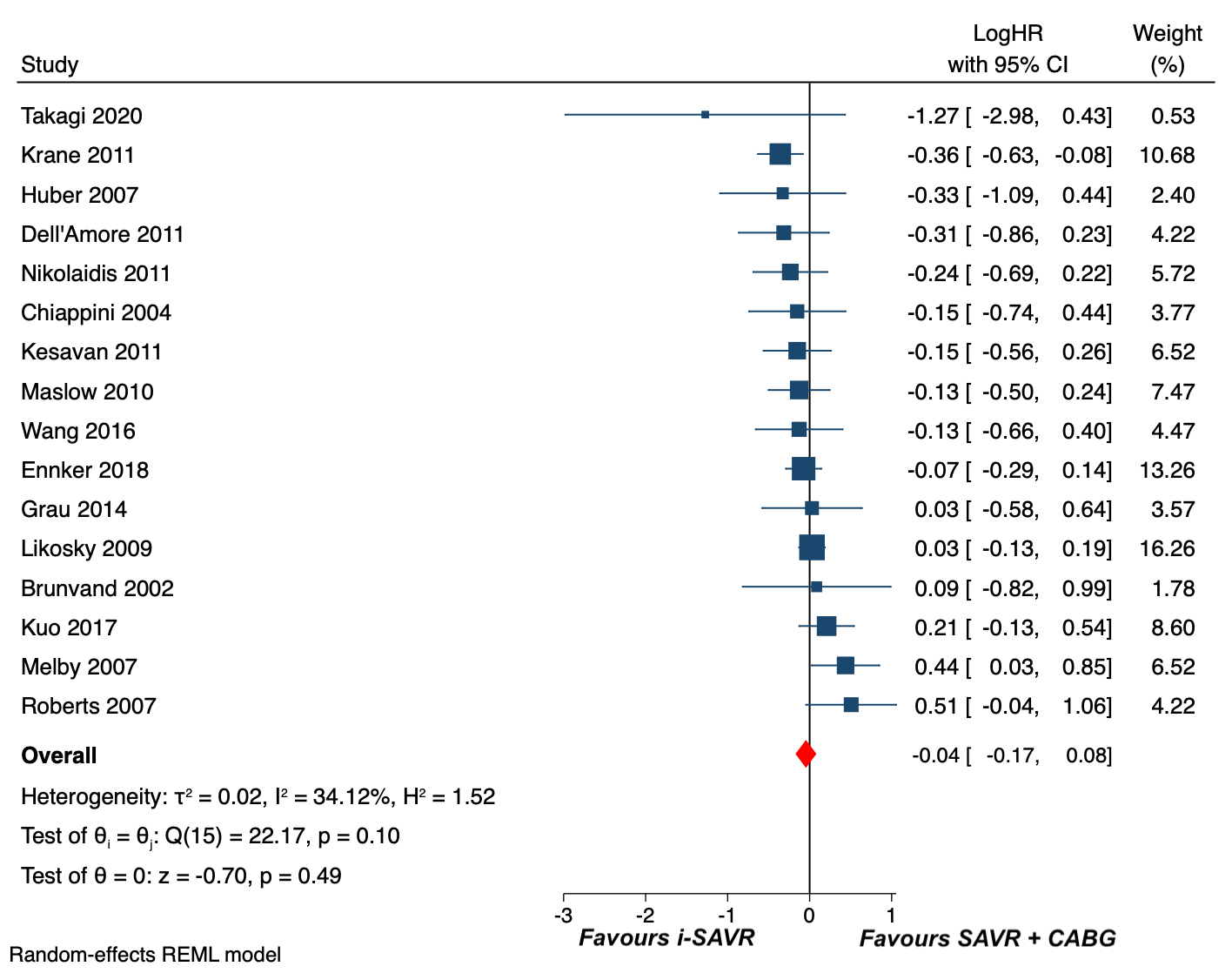


**Figure S7.** Additional sensitivity analysis using effect estimates based on logHR.

*HR, hazard ratio; i-SAVR, isolated aortic valve replacement; CABG, coronary artery bypass grafting.*


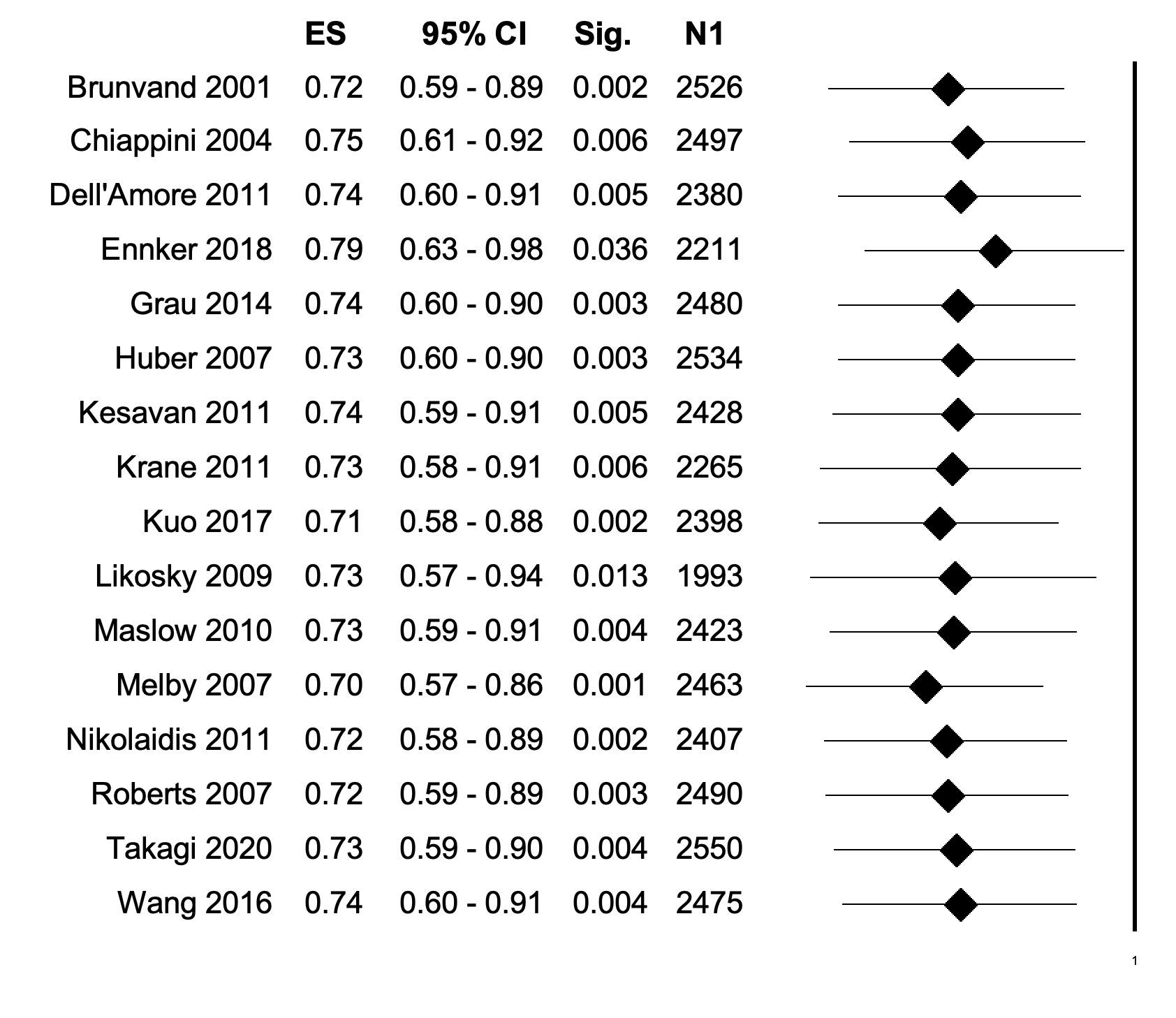


**Figure S8**. The sensitivity analysis according to the leave-one-out method to identify any influential studies on the pooled data for early mortality.

*HR, hazard ratio; CI, confidence interval; Sig, P value.*


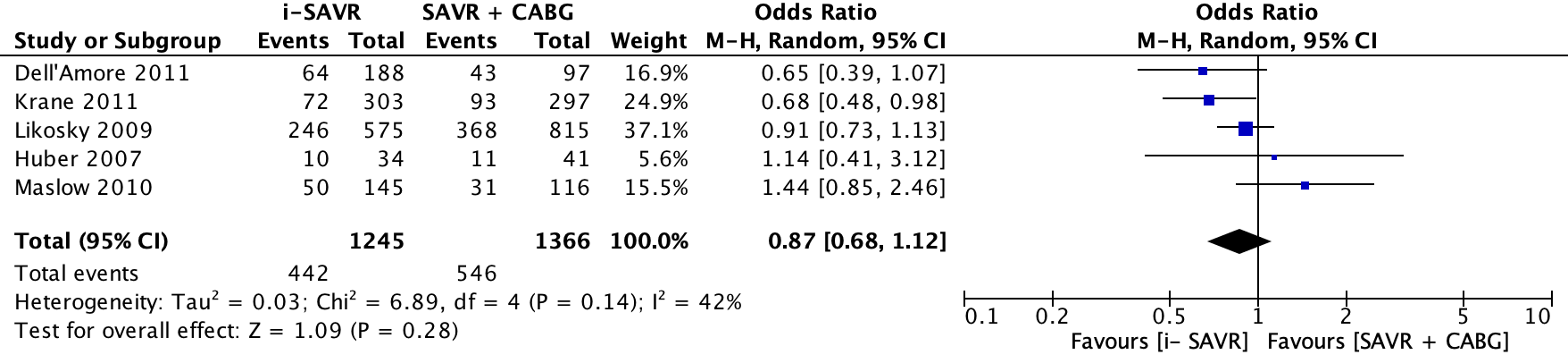


**Figure S9**. Forest plot for new onset postoperative atrial fibrillation. No difference was observed between isolated aortic valve replacement (i-SAVR) and SAVR + coronary artery bypass grafting (CABG). I^2^, 42% indicates moderate heterogeneity.

*HR, hazard ratio; CI, confidence interval; W, weight; Sig, P value.*


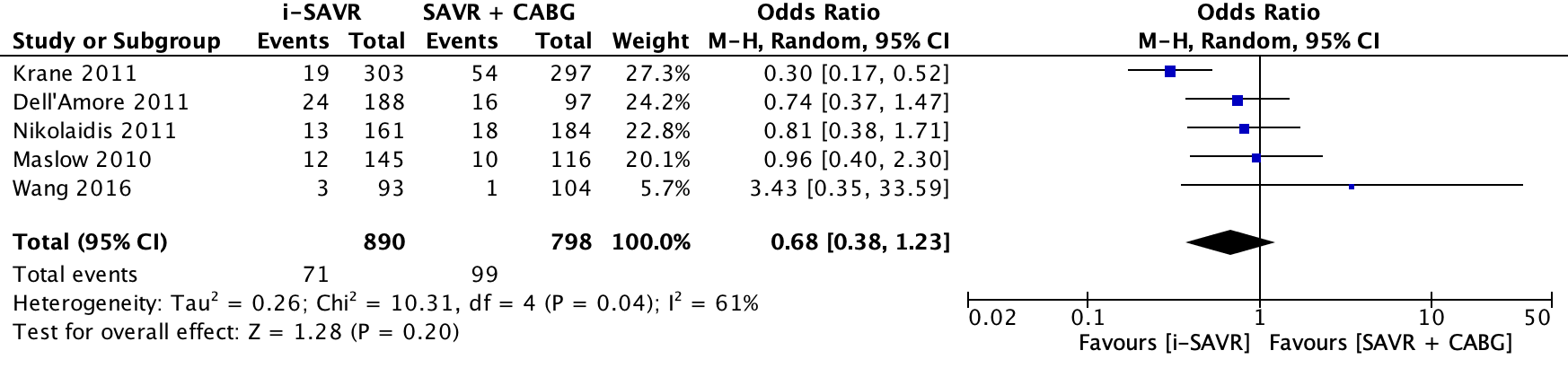


**Figure S10**. Forest plot for postoperative acute renal failure. No difference was observed between isolated aortic valve replacement (i-SAVR) and SAVR + coronary artery bypass grafting (CABG). I^2^, 61% indicates significative heterogeneity.

*HR, hazard ratio; CI, confidence interval; W, weight; Sig, P value.*


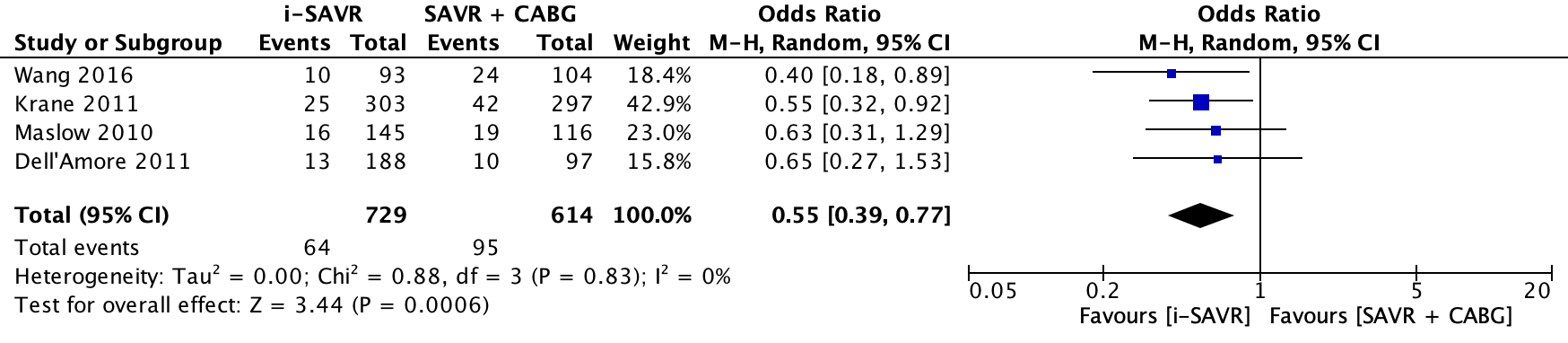


**Figure S11**. Forest plot for prolonged mechanical ventilation. No difference was observed between isolated aortic valve replacement (i-SAVR) and SAVR + coronary artery bypass grafting (CABG). I^2^, 0% indicates no evidence of heterogeneity.

*HR, hazard ratio; CI, confidence interval; W, weight; Sig, P value.*


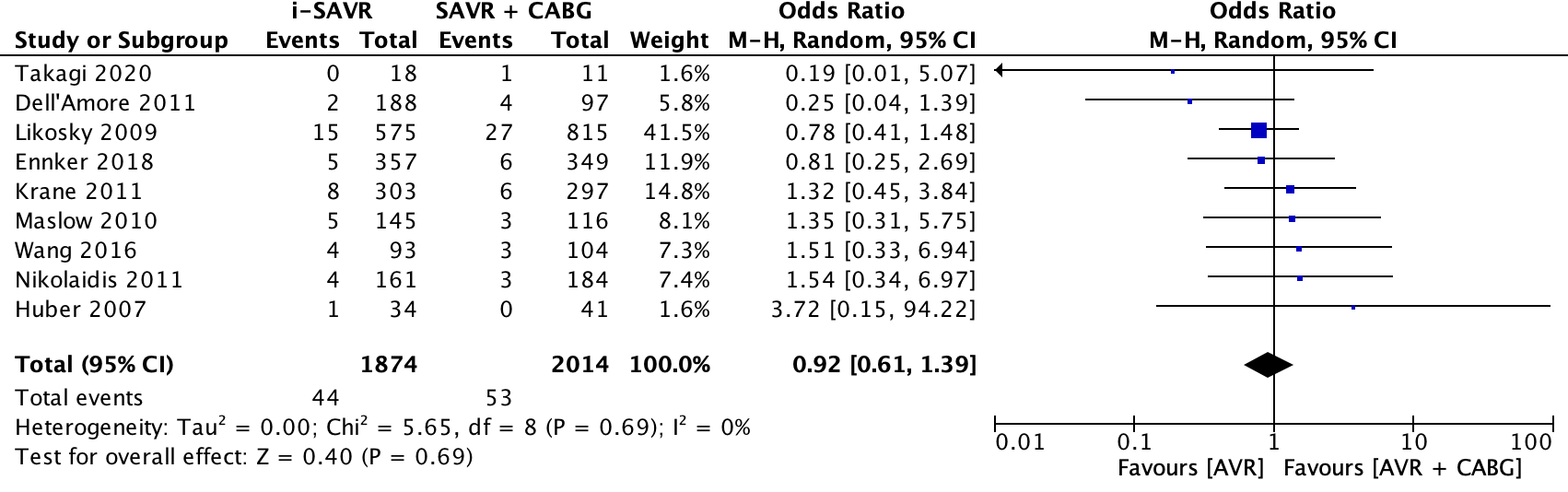


**Figure S12**. Forest plot for postoperative cerebrovascular events. No difference was observed between isolated aortic valve replacement (i-SAVR) and SAVR + coronary artery bypass grafting (CABG). I^2^, 0% indicates no evidence of heterogeneity.

HR, hazard ratio; CI, confidence interval; W, weight; Sig, *P* value.


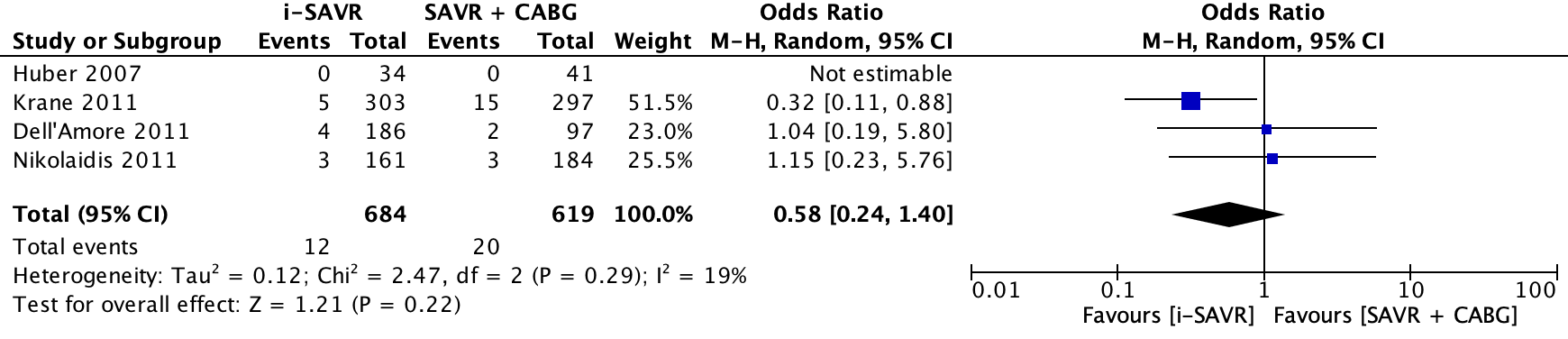


**Figure S13**. Forest plot for postoperative intra-aortic balloon pump. No difference was observed between isolated aortic valve replacement (i-SAVR) and SAVR + coronary artery bypass grafting (CABG). I^2^, 19% indicates low heterogeneity.

*HR, hazard ratio; CI, confidence interval; W, weight; Sig, P value.*


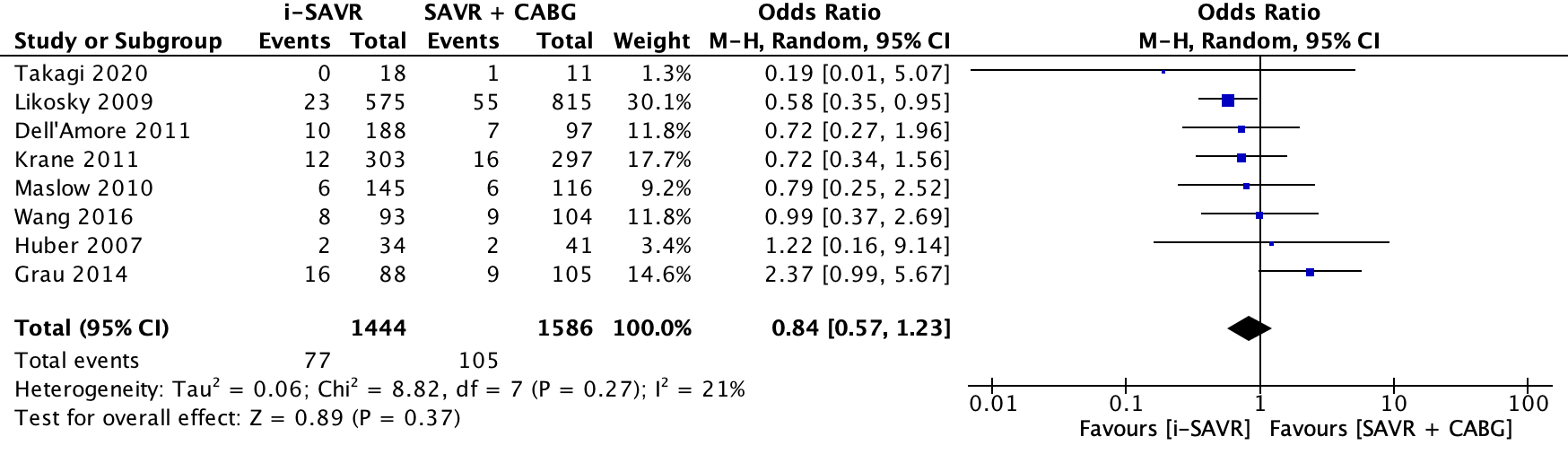


**Figure S14**. Forest plot for re-thoracotomy for bleeding/tamponade. No difference was observed between isolated aortic valve replacement (i-SAVR) and SAVR + coronary artery bypass grafting (CABG). I^2^, 21% indicates low heterogeneity.

*HR, hazard ratio; CI, confidence interval; W, weight; Sig, P value.*


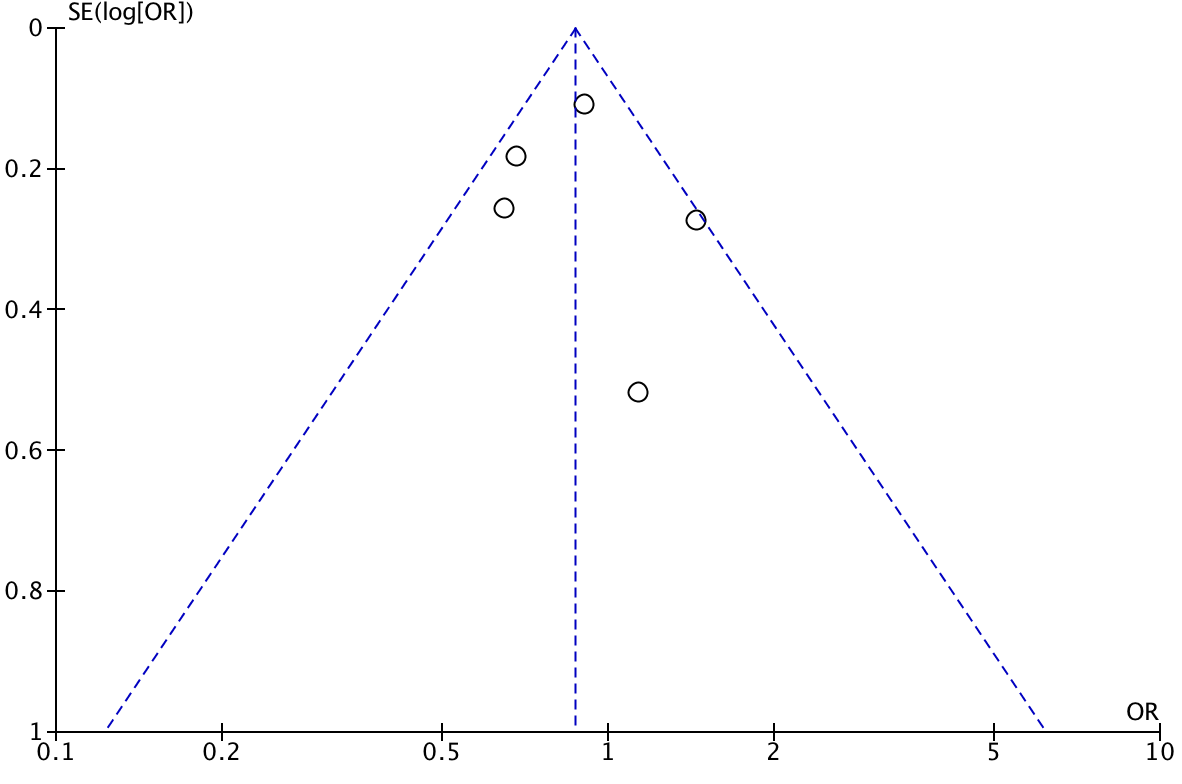


**Figure S15**. Funnel plot to assess publication bias. No publication bias was reported related to postoperative atrial fibrillation. Egger’s test, p = 0.99


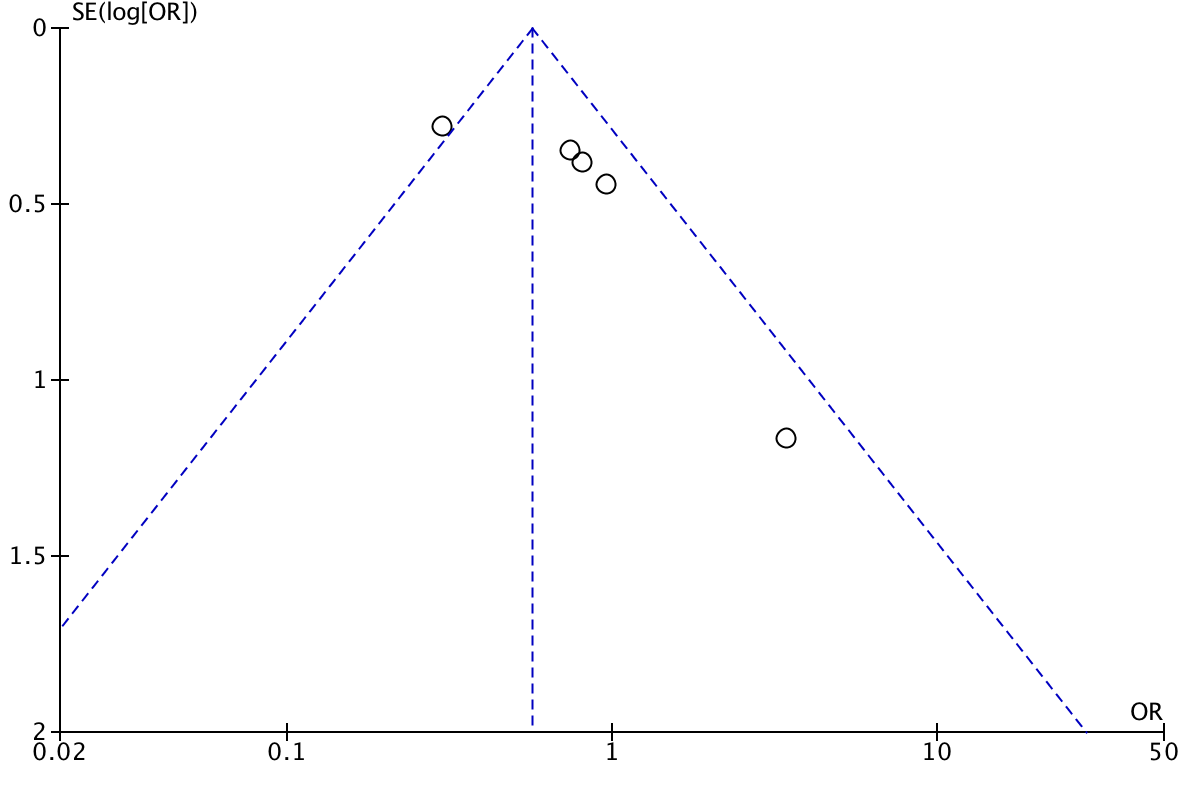


**Figure S16**. Funnel plot to assess publication bias. No publication bias was reported related to postoperative acute renal failure. Egger’s test, p = 0.11


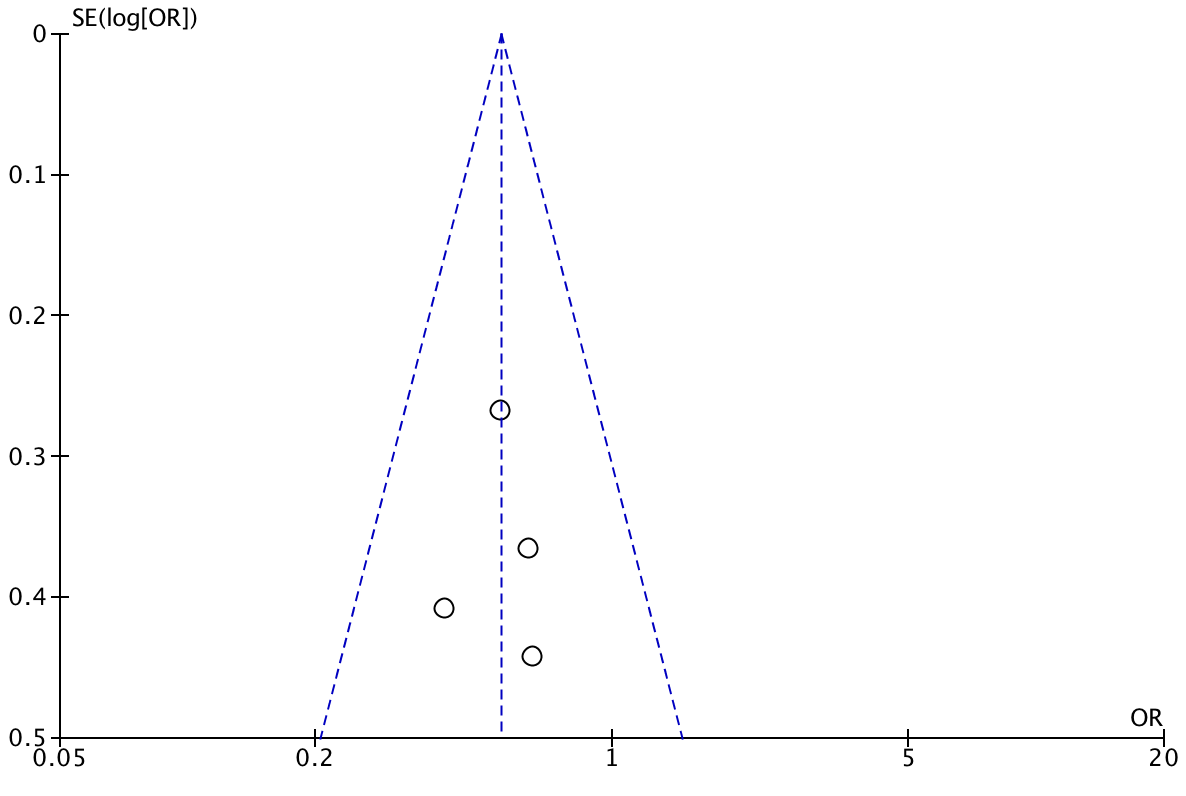


**Figure S17**. Funnel plot to assess publication bias. No publication bias was reported related to prolonged mechanical ventilation. Egger’s test, p = 0.97


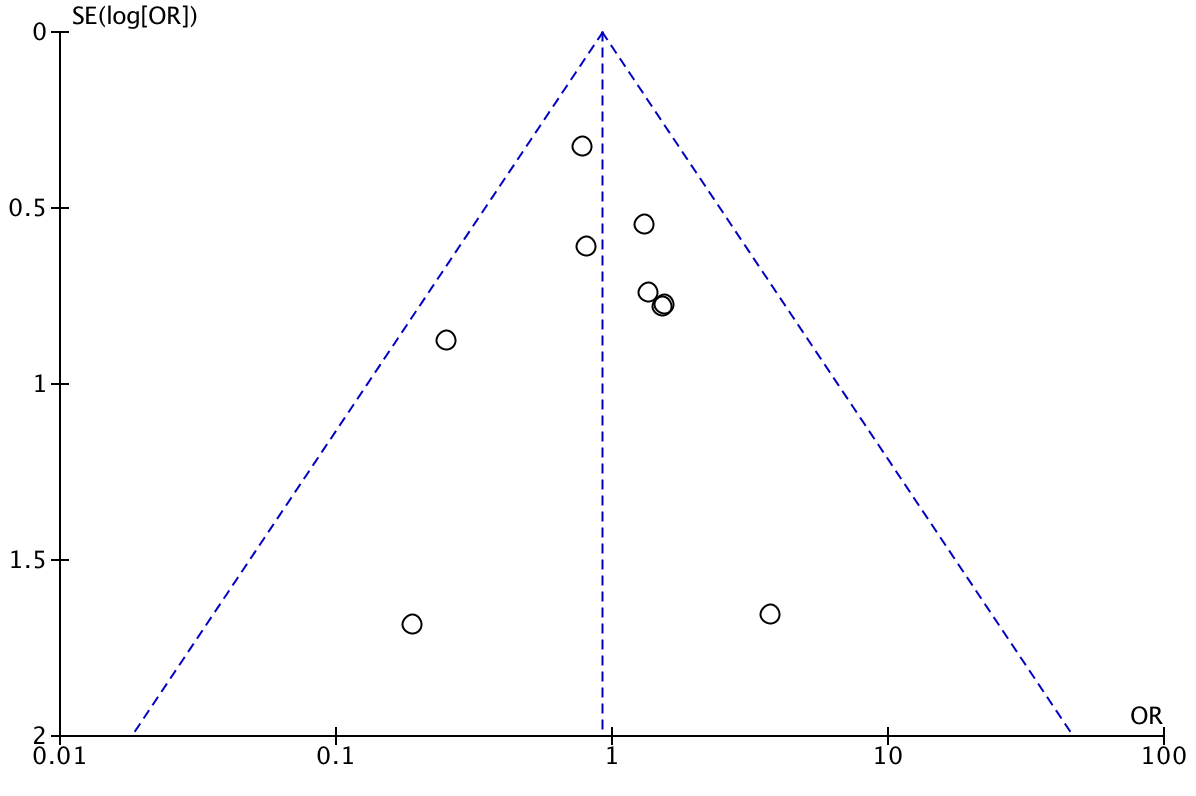


**Figure S18**. Funnel plot to assess publication bias. No publication bias was reported related to postoperative cerebrovascular events. Egger’s test, p = 0.72


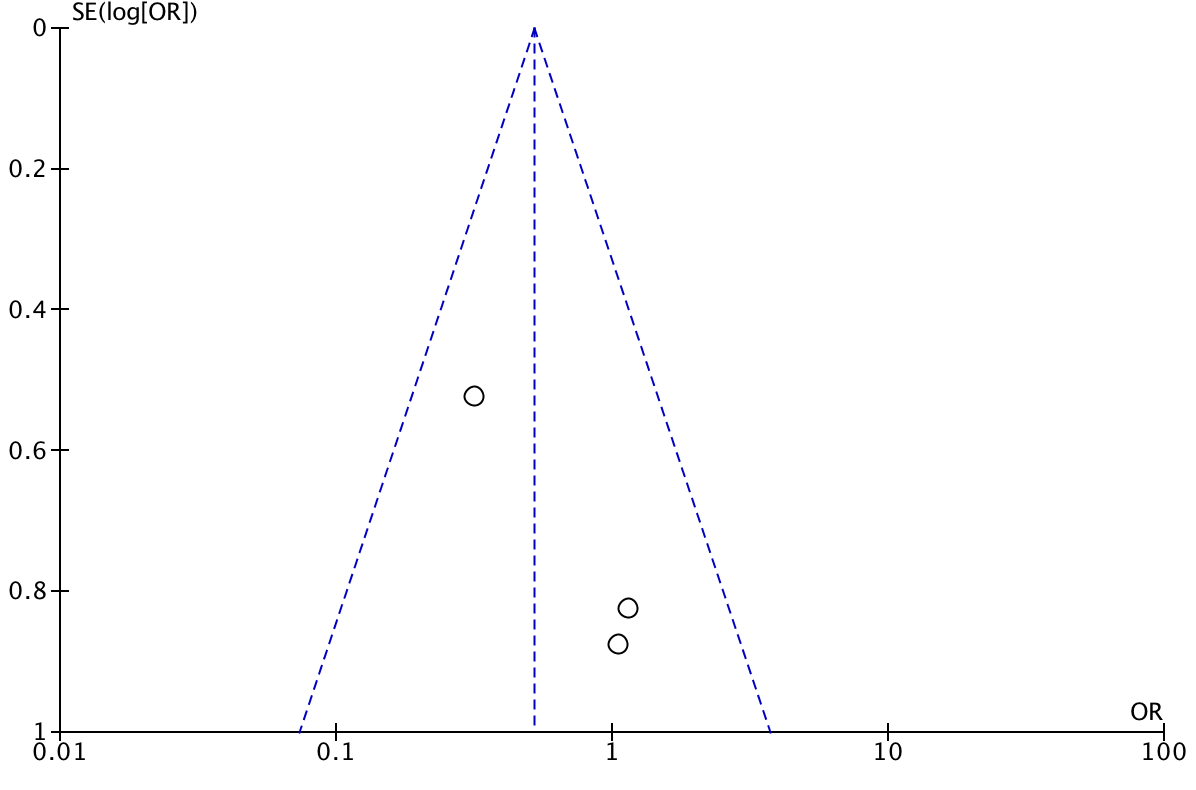


**Figure S19**. Funnel plot to assess publication bias. No publication bias was reported related to postoperative intra-aortic balloon pump. Egger’s test, p = 0.09


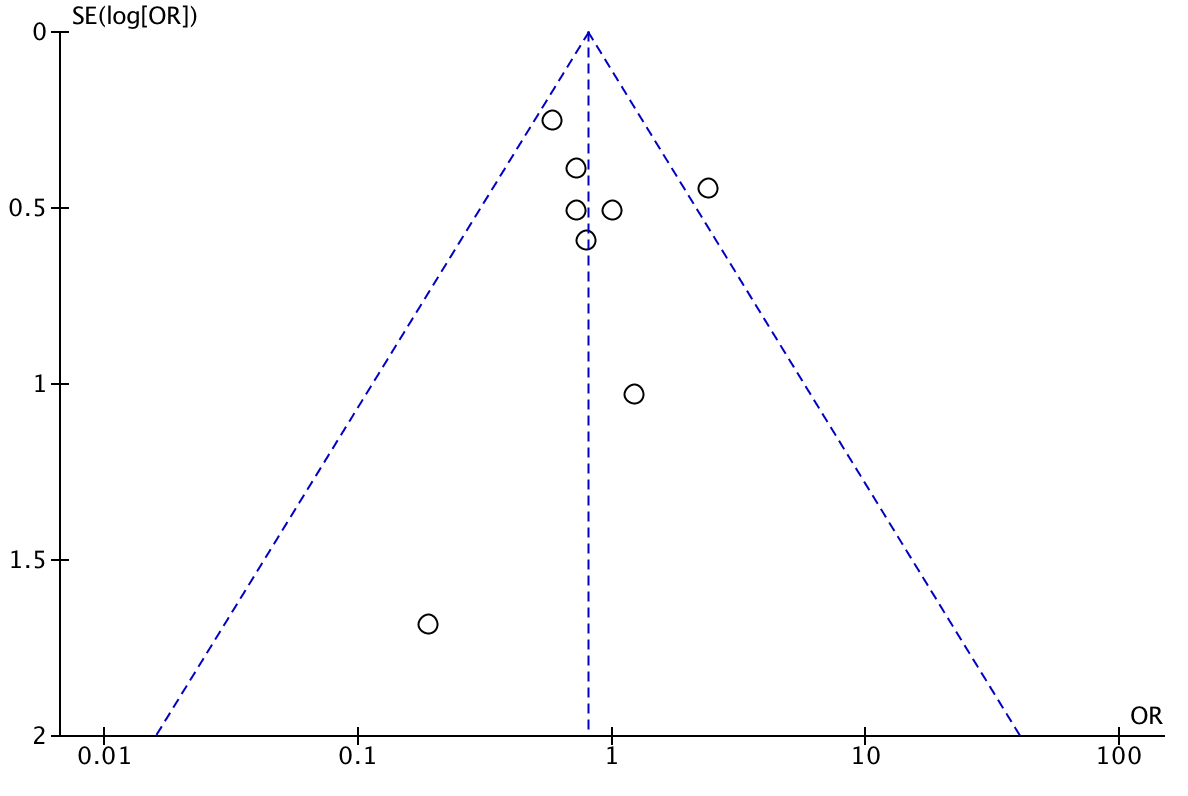


**Figure S20**. Funnel plot to assess publication bias. No publication bias was reported related to re-thoracotomy for bleeding/tamponade. Egger’s test, p = 0.79
